# Integrating artificial intelligence to assess emotions in learning environments: a systematic literature review

**DOI:** 10.3389/fpsyg.2024.1387089

**Published:** 2024-06-19

**Authors:** Angel Olider Rojas Vistorte, Angel Deroncele-Acosta, Juan Luis Martín Ayala, Angel Barrasa, Caridad López-Granero, Mariacarla Martí-González

**Affiliations:** ^1^Psychology Department, European University of the Atlantic, Santander, Spain; ^2^Psychology Department, International Ibero-American University, Mexico, Mexico; ^3^Escuela de Postgrado, Universidad San Ignacio de Loyola, Lima, Peru; ^4^Department of Psychology and Sociology, University of Zaragoza, Teruel, Spain; ^5^Department of Social Anthropology and Social Psychology, Complutense University of Madrid, Madrid, Spain

**Keywords:** emotions, artificial intelligence, teaching-learning, education, assessment

## Abstract

**Introduction:**

Artificial Intelligence (AI) is transforming multiple sectors within our society, including education. In this context, emotions play a fundamental role in the teaching-learning process given that they influence academic performance, motivation, information retention, and student well-being. Thus, the integration of AI in emotional assessment within educational environments offers several advantages that can transform how we understand and address the socio-emotional development of students. However, there remains a lack of comprehensive approach that systematizes advancements, challenges, and opportunities in this field.

**Aim:**

This systematic literature review aims to explore how artificial intelligence (AI) is used to evaluate emotions within educational settings. We provide a comprehensive overview of the current state of research, focusing on advancements, challenges, and opportunities in the domain of AI-driven emotional assessment within educational settings.

**Method:**

The review involved a search across the following academic databases: Pubmed, Web of Science, PsycINFO and Scopus. Forty-one articles were selected that meet the established inclusion criteria. These articles were analyzed to extract key insights related to the integration of AI and emotional assessment within educational environments.

**Results:**

The findings reveal a variety of AI-driven approaches that were developed to capture and analyze students’ emotional states during learning activities. The findings are summarized in four fundamental topics: (1) emotion recognition in education, (2) technology integration and learning outcomes, (3) special education and assistive technology, (4) affective computing. Among the key AI techniques employed are machine learning and facial recognition, which are used to assess emotions. These approaches demonstrate promising potential in enhancing pedagogical strategies and creating adaptive learning environments that cater to individual emotional needs. The review identified emerging factors that, while important, require further investigation to understand their relationships and implications fully. These elements could significantly enhance the use of AI in assessing emotions within educational settings. Specifically, we are referring to: (1) federated learning, (2) convolutional neural network (CNN), (3) recurrent neural network (RNN), (4) facial expression databases, and (5) ethics in the development of intelligent systems.

**Conclusion:**

This systematic literature review showcases the significance of AI in revolutionizing educational practices through emotion assessment. While advancements are evident, challenges related to accuracy, privacy, and cross-cultural validity were also identified. The synthesis of existing research highlights the need for further research into refining AI models for emotion recognition and emphasizes the importance of ethical considerations in implementing AI technologies within educational contexts.

## 1 Introduction

The integration of Artificial Intelligence (AI) into educational settings marks a significant advancement in detecting, assessing, and nurturing students’ emotions. AI’s ability to analyze complex emotional behavior patterns through data collected during the learning process enables a deeper understanding of each student’s needs.

By employing advanced algorithms, AI can detect signs of frustration, boredom, or enthusiasm, allowing educators to tailor their teaching methods more effectively. Additionally, AI can provide instant, personalized feedback based on emotional analysis, thereby creating a learning environment that is more attuned to students’ emotional well-being. This comprehensive approach significantly contributes to students’ holistic development, enhancing their ability to manage emotions, build positive relationships, and improve their academic performance.

In this regard D’Mello and Graesser (2012) raise that AI can predict student emotions (boredom, fluency/engagement, confusion, and frustration) by analyzing the text of dialogues between students and tutors during interactions with an “Intelligent Tutoring System.” These AI-driven intelligent tutoring systems can positively influence student motivation by incorporating artificially intelligent educational models, such as the “Mobile Adaptive Personalized Learning Environment” -MAPLE- (Mehigan and Pitt, 2019). Thus, artificial tutors with synthesized emotions can adapt their behavior to students’ reactions and affective states, improving their performance in e-learning systems (Florea and Kalisz, 2005).

Another interesting study argues that AI can help detect and assess students’ emotions within interactive digital learning environments (IDLE) and adapt the environment accordingly to meet their real needs, potentially improving learning (Arguel et al., 2019). AI may also classify students’ emotions during their interaction with immersive environments, allowing for a better understanding of their emotional experiences (Rodríguez et al., 2020).

AI can also analyze emotions from text, enhancing student motivation and performance in e-learning environments (Rodriguez et al., 2012). Simultaneously, it can gauge the intensity of emotions and tailor lessons to individual needs, promoting successful completion of academic studies (Sumithra et al., 2022). Similarly, a recent study found that using deep learning methods to detect students’ emotions can significantly boost productivity and enhance the educational process (AlZu’bi et al., 2022).

In a systematic review, de Oliveira and Rodrigues (2021) discovered that 60% of recent studies on human behavior and AI, specifically from the past three and a half years, focus on emotion-driven organizations. This trend highlights the growing interest and novelty of the field.

Among the efforts to incorporate AI into emotional management within educational settings, the “Biologically Inspired Cognitive Architecture” (eBICA) is notable. Developed by Samsonovich (2020), eBICA allows AI to understand and interact with human emotions during social interactions. Additionally, the emotion-based artificial decision-making model has been shown to enhance the performance of educational agents in virtual settings (Yang and Zhen, 2014). Another approach involves the integration of emotional agents in AI-based learning environments to improve learner motivation, self-assessment, and self-motivation by improving the socioemotional climate (Gorga and Schneider, 2009), especially affective computing (Kort et al., 2001; González-Hernández et al., 2018; Ninaus et al., 2019; Shobana and Kumar, 2021; He et al., 2022; Aly et al., 2023; Villegas-Ch et al., 2023).

Recent advancements reveal that artificial intelligence (AI) can not only recognize but also predict emotions (Alm et al., 2005; Lin et al., 2023; Singh et al., 2023). This capability extends beyond identifying current emotional states, enabling systems such as virtual assistants and Intelligent Tutoring Systems (ITS) to proactively adapt and respond more effectively to students’ emotional needs, thus enhancing the learning experience.

AI also significantly impacts social emotions such as empathy, compassion, and interpersonal phenomena like justice and cooperation, which are crucial for learning (Lamm and Singer, 2010).

Furthermore, AI can analyze empathic behavior in dynamic social contexts like educational settings. There are now models that use deep learning to foster emotional intelligence, processing multimodal emotional signals to generate appropriate empathic responses (Alanazi et al., 2023).

Overall, despite the challenges associated with AI’s empathic abilities, it is acknowledged that AI offers valuable tools for promoting empathic skills, essential for social cooperation, and ethical and prosocial behavior (Gómez-León, 2022).

The importance of AI in supporting mental health is finally recognized, an area supported by hundreds of progressively increasing studies (Mohr et al., 2017; Garcia-Ceja et al., 2018; Graham et al., 2019; Shatte et al., 2019) taking into account that AI systems can provide emotional support and personalized advice to students and other educational actors experiencing stress or depression and provide advice and feedback based on emotional well-being.

### 1.1 Intelligent tutoring systems and emotions

Intelligent Tutoring Systems (ITS) are closely related to students’ emotions, since learning and emotions are an inseparable binomial. This is expressed in the cognitive-affective unity of the human personality. Intelligent tutoring systems are evolving to address not only the cognitive aspect of learning, but also the emotional needs of students to improve their educational experience and performance. In this sense, configurations are being incorporated that enable ITS to detect emotions, content adaptation, emotional support, and personalized feedback, moving toward an emotionally intelligent tutoring system (Mohanan et al., 2018).

A study involving “MetaTutor,” a hypermedia-based intelligent tutoring system (ITS), showcases the capabilities of ITS to enhance learning experiences. MetaTutor provides students with feedback on the impact of positive and negative emotions during learning. It also guides students on how to regulate specific emotions to optimize learning effectiveness. Importantly, MetaTutor assesses not only cognitive processes but also metacognitive processes, emphasizing its comprehensive approach to student learning and emotional management (Taub et al., 2021).

A review study on emotion regulation in intelligent tutoring systems (ITS) highlights a consensus among researchers in computerized learning. It suggests that ITS could greatly enhance their effectiveness if they were able to adapt to the emotional states of students. This adaptation would allow ITS to better support personalized learning experiences by responding dynamically to the emotional and cognitive needs of each student (Malekzadeh et al., 2015).

There is a growing body of research linking intelligent tutoring systems (ITS) to emotion during the learning process. Among the most significant advances is the analysis of facial expressions to estimate the emotional state of a student using ITS (Sarrafzadeh et al., 2003); the relationship between emotion variability, self-regulated learning and task performance in ITS (Li S. et al., 2021; Li W.-C. et al., 2021); inducing positive emotional states in ITS (Chaffar et al., 2009); a new approach toward model students’ socio-emotional attributes to predict their performance in ITS (Assielou et al., 2021); the integration of emotion management strategies in ITS (Malekzadeh et al., 2014); emotional pedagogical agents in ITS (Sun et al., 2013); the use of emotional coping strategies in ITS (Chaffar and Frasson, 2010); among many other results that clearly show that ITS have a close link with human emotions.

## 2 Methods and procedures

We performed a systematic review of the scientific literature through the following databases: Pubmed, Web of Science, PsycINFO and Scopus. These articles were analyzed to extract key insights related to the integration of AI and emotional assessment within educational environments. Additionally, reference lists of included studies and reviews were checked for potentially relevant articles not identified through the electronic search.

The identification of thematic clusters was carried out through a process of analysis and synthesis of the studies included in the review. The criteria used were the following:

1. *Thematic frequency*: This criterion allowed us to identify the frequency with which certain themes or concepts appeared in the studies reviewed. This involved searching for and recording patterns of key terms in the titles, abstracts, keywords and sections of the studies reviewed.
2. *AI technology used*: This criterion is based on the specific artificial intelligence technologies used in the studies reviewed. It involves a detailed analysis of the techniques, tools, algorithms and technological approaches used for the evaluation of emotions in educational environments.
3. *Domain or scope of application*: This criterion focused on the specific contexts in which artificial intelligence technologies were applied to evaluate emotions in educational settings, including special education. It examines whether the studies focused on particular areas such as general education, vocational training or distance learning, as well as special education for students with special educational needs.
4. *Results*: This criterion allowed us to examine the findings of each study, especially in relation to the relevant aspects for the integration of artificial intelligence in the evaluation of emotions in educational environments. The observed effects, conclusions reached and implications for educational practice were considered.

Once these criteria have been determined, we continue with the process of identifying thematic clusters, following the following seven-step procedure:

1. *Study selection*: We began with an exhaustive search of the relevant literature using academic databases and specialized search engines. Predefined inclusion and exclusion criteria were applied to select relevant studies that addressed the topic of integrating artificial intelligence to assess emotions in educational settings.
2. *Information extraction*: Based on the established criteria, the research team began the process of extracting key information from each selected study, such as recurring concepts and processes; Applied AI technology, intervention context, results and main conclusions. This information provided a solid basis for analysis and comparison between studies.
3. *Identification of emerging themes*: All the extracted information was examined to identify recurring themes and organize emerging patterns related to the integration of artificial intelligence and the evaluation of emotions in educational environments. This involved a rereading of each study with the extracted information to understand its content and context.
4. *Data Coding*: Codes or labels were assigned to each emerging theme or pattern.
5. *Grouping into thematic clusters*: Using the codes assigned to each study or fragment, the codes were grouped into coherent thematic clusters. This process involved identifying similarities and relationships between the coded information and organizing them into groups that address specific aspects of integrating artificial intelligence to assess emotions in educational settings.
6. *Refinement and validation*: Thematic clusters were reviewed and refined to ensure consistency and relevance. Cross-validation was carried out between the researchers involved in the review to ensure accuracy and consistency in the grouping of studies. At first, 9 clusters had been formed, however, this process allowed for greater integration, managing to refine and achieve 4 thematic clusters.
7. *Analysis and synthesis*: Once thematic clustering was completed, a detailed analysis of the studies within each cluster was conducted to identify trends, discrepancies, and notable areas of interest. This stage allowed us to synthesize the information collected and provide a contextualized view of the literature reviewed on the topic.

These thematic clusters were organized with the objective of providing a coherent structure to analyze and synthesize the information collected, thus facilitating the understanding of trends and advances in the integration of artificial intelligence to evaluate emotions in educational environments.

**Figure 1:**
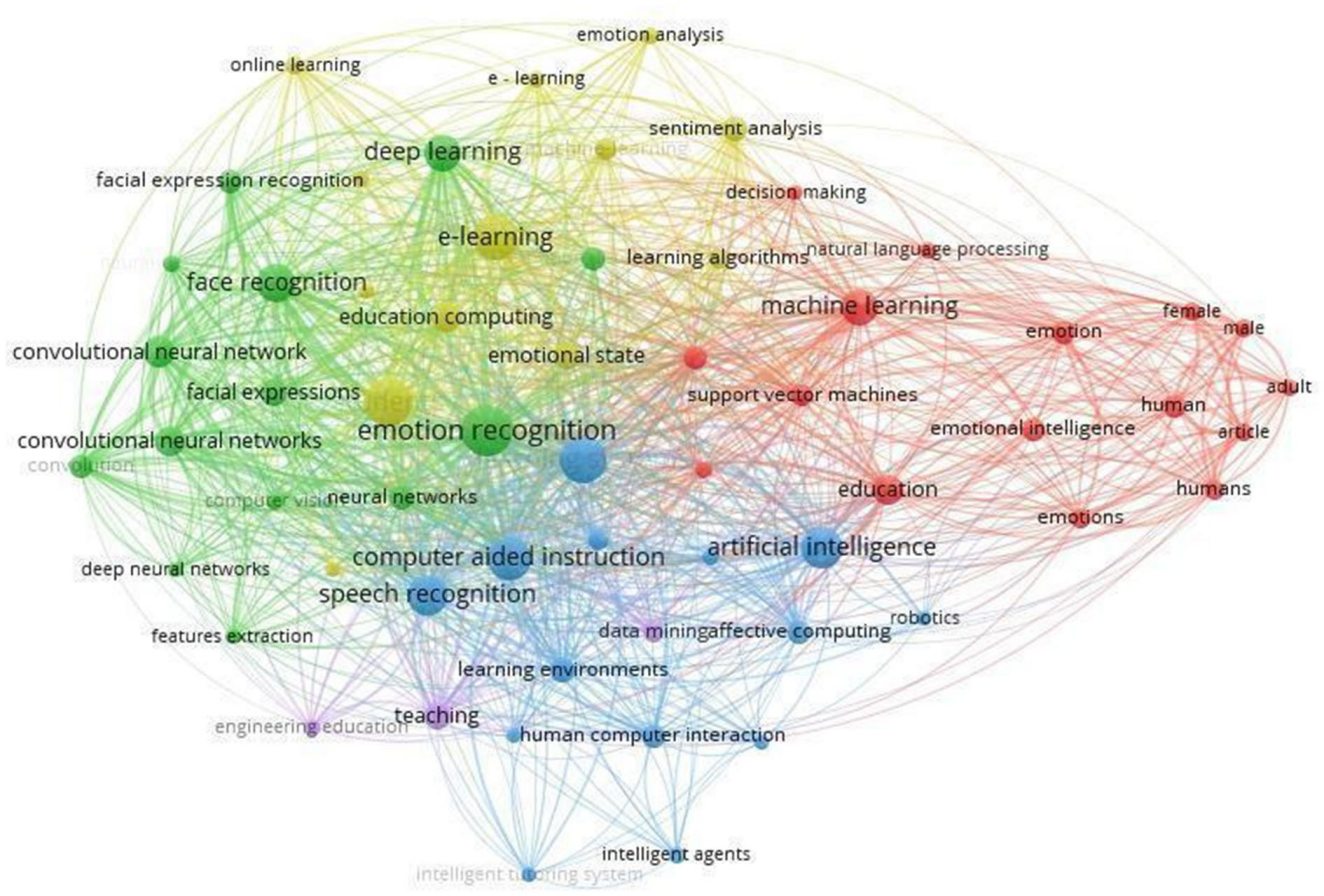
Co-occurrence network. VOSviewer_1.6.18.

We used the following search terms: artificial intelligence terms AND recognition of emotions AND educational context terms as follows (Figure 1):

**Cluster 1**: “artificial intelligence” OR “machine intelligence” OR “intelligent support” OR “intelligent virtual reality” OR “chat bot*” OR “machine learning” OR “automated tutor” OR “personal tutor*” OR “intelligent agent*” OR “expert system” OR “neural network” OR “natural language processing.”

**Cluster 2**: “Emotion recognition” OR “Speech Emotion Recognition” OR “Emotion Classification” OR “Emotional State” OR “Facial Emotion Recognition” OR “Facial Emotions” OR “Emotion Detection” OR “Emotionality” OR “Human Emotion” OR “Emotional Speech” OR “Multimodal Emotion Recognition” OR “Emotional Intelligence” OR “Automatic Emotion Recognition” OR “Human Emotion Recognition” OR “Emotion Analysis.”

**Cluster 3**: “educational” OR “educational environments” OR “learning environments” OR “Educational Settings” OR “educational context” OR “pedagogical environments” OR “academic settings” OR “classroom environments” OR “learning spaces” OR “educational institutions” OR “school environments” OR “educational facilities” OR “teaching and learning environments” OR “educational institutions” OR “school systems” OR “academic programs” OR “higher education” OR “pedagogical approaches” OR “university campuses.”

Titles and abstracts were screened, and full reports of potentially relevant studies were obtained using a Preferred Reporting Items for Systematic reviews and Meta-Analyses (PRISMA) model (Page et al., 2021). Two authors (AORV and AD) independently assessed the reports for eligibility, with discrepancies resolved by discussion with a third author (JL).

We included quantitative studies in English, Spanish, and Portuguese, and studies related to both virtual and face-to-face educational environments. Articles were excluded based on the following exclusion criteria: (1) if they referred to non-data-based studies (e.g., editorials, commentaries, opinion papers, and review papers), and (2) if stigmatizing attitudes were assessed among non-physician primary care professionals, such as nurses, technicians, social workers, and other professionals, among mental health professionals, or among the general population. Data on study design, sample characteristics, and findings were extracted independently by three authors (MC, CAF and JLMA). Because of the heterogeneity between studies, which hindered a statistical synthesis of their results, we summarized evidence from articles included in the review through a narrative synthesis (Popay et al., 2006).

## 3 Results and discussion

**Figure 2:**
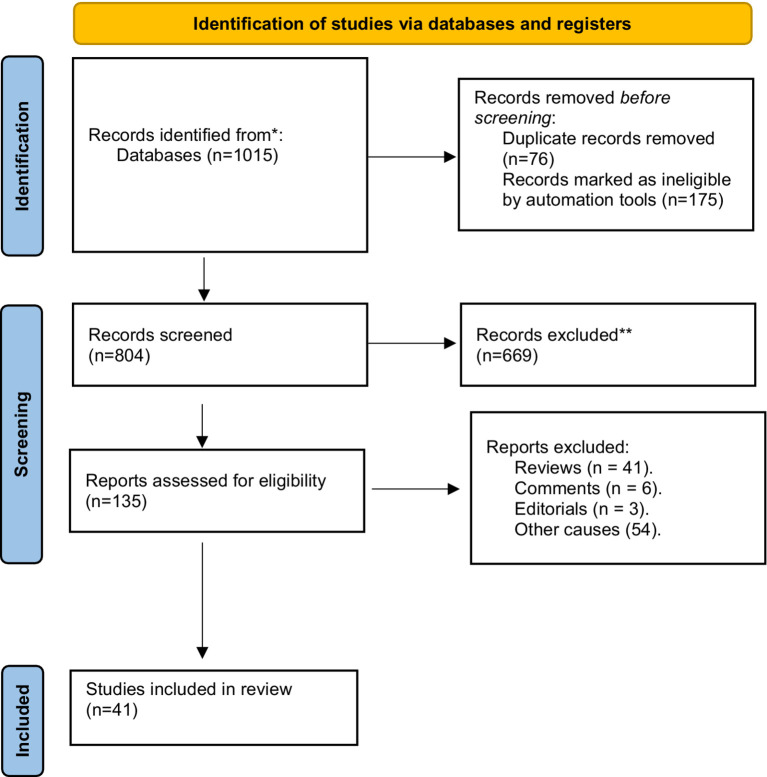
The number of articles found to be included in the review (Page et al., 2021).

One thousand and fifteen articles were identified in the four databases (Scopus: 366, Web of Science: 203, Pubmed: 163 and PsycINFO: 283). 135 articles were identified as potentially relevant and were assessed against eligibility criteria. Forty-one studies fulfilled inclusion criteria using the PRISMA model (Figure 2) and are summarized in Supplementary Table S1.

Every study included in the review used a cross-sectional design or used databases to investigate the integration of artificial intelligence (AI) for emotional assessment within educational contexts. Nine studies were conducted in China, one in Colombia, Ecuador, Egypt, Germany, seven in India, two in Iran, one in Japan and Jordan, two in Morocco, two in Russia and Spain, one in Thailand, Tunisia and United Arab Emirates, and five in the United States.

Based on the results of the articles included in the review, we can consider that there are several points in common and others that are more specific. We present the results and discussion based on each of these topics: *emotion recognition in education, technology integration and learning outcomes, special education and assistive technology, affective computing.*

### 3.1 Emotion recognition in education

Emotion recognition is essential for understanding how emotions affect peer interactions, academic performance, and engagement in online and virtual learning environments (Standen et al., 2020; Dehbozorgi and Kunuku, 2023; Villegas-Ch et al., 2023).

The main methods used in the research to analyze students’ emotional states were related to facial expressions, eye movements, and biosignal data (Ninaus et al., 2019; Dehbozorgi and Kunuku, 2023; Villegas-Ch et al., 2023; Yugal et al., 2023). During online lessons, monitoring systems studied real-time attention, emotions and feelings (He et al., 2022; Dehbozorgi and Kunuku, 2023).

In educational settings, the use of artificial intelligence, particularly machine learning and deep learning, has grown increasingly popular. These technologies primarily enhance the speed of analysis and the accuracy of emotion classification (He et al., 2022; Begum et al., 2023; Villegas-Ch et al., 2023; Yugal et al., 2023). Although artificial intelligence has seen significant advancements recently, various models have also been employed for speech emotion recognition to explore the relationship between emotions and academic performance (Dehbozorgi and Kunuku, 2023).

In this sense, numerous studies have performed detailed analyses to uncover the relationship between students’ expressed emotions and their academic performance (Dehbozorgi and Kunuku, 2023; Yugal et al., 2023). Positive emotions like relief and satisfaction are strongly correlated with higher grades, suggesting that students experiencing these emotions typically achieve better academically. Conversely, negative emotions, such as frustration, are negatively correlated with academic performance, indicating that students experiencing these emotions often face academic challenges.

Positive emotional states have been associated with greater success in completing class activities on time and better overall performance, underscoring the importance of emotional well-being in academic settings (Dehbozorgi and Kunuku, 2023).

These technologies have been instrumental in identifying the impact of emotions on learning outcomes, linking positive emotions to improved cognitive processes and engagement (He et al., 2022; Villegas-Ch et al., 2023; Yugal et al., 2023).

### 3.2 Technology integration and learning outcomes

In the current educational landscape, integrating technology is key to enhancing learning outcomes. Blending technological tools with traditional teaching methods has created new opportunities to enrich the educational experience and foster skill development in students. The effectiveness of this integration is evident in its adaptability to various learning styles, its ability to boost student engagement, and its role in providing access to global educational resources.

In the context of our research on using artificial intelligence to assess emotions in learning environments, it is essential to understand how this synergy between technology and learning outcomes can improve the educational process and make achieving learning objectives more effective and meaningful.

Technologies such as AI, machine learning, and deep learning are employed to expedite emotion analysis and enhance classification accuracy in educational settings (He et al., 2022; Dehbozorgi and Kunuku, 2023). The integration of artificial intelligence (AI) into the management of emotions within education marks a significant advancement in modern teaching methods. Research has shown that machine learning techniques can reliably identify a range of human emotions, including happiness, anger, sadness, and calmness (Ramirez and Vamvakousis, 2012).

This ability can significantly enhance teaching by providing a deeper, more personalized understanding of students’ emotional states. Such insights allow for the customization of teaching strategies to better address individual needs. Effectively applying AI in managing educational emotions can not only boost students’ overall well-being but also foster a more inclusive and empathetic learning environment.

Improved emotional recognition from “EEG signals” can be enhanced by integrating deep learning with shallow machine learning techniques, which holds promising applications in human-computer interaction (Islam et al., 2021). This development signifies a major research advancement, recognizing deep learning’s ability to extract complex features from EEG signals and the role of shallow machine learning in providing a clearer, more interpretable analysis. Combining these approaches creates a synergistic effect, enhancing the detection and understanding of emotions from EEG signals. Such advancements could lead to innovative applications in human-computer interaction, resulting in more intuitive and adaptive interfaces that align with users’ emotions and needs.

Artificial intelligence-based educational models, like “MAPLE,” are poised to positively influence student motivation and engagement in e-learning environments by catering to their emotional needs (Mehigan and Pitt, 2019). This underscores the value of adaptability and personalization in these systems, allowing for more targeted responses to learners’ emotional states. By incorporating artificial intelligence, educational environments become more responsive and empathetic, thereby enhancing student engagement and satisfaction.

Another significant development is the emergence of affective computing and sentiment analysis. These fields utilize human-computer interaction, information retrieval, and multimodal signal processing to analyze sentiments from online social data, providing valuable insights for educational sciences (Cambria, 2016; Cambria et al., 2017). These advancements facilitate a deeper understanding of emotional experiences in digital settings, which can inform both online and offline educational strategies. Integrating these emotional analytics into education enhances the customization of teaching methods and curriculum design to better meet students’ emotional needs, promoting more effective and meaningful learning experiences.

#### 3.2.1 Emotionally intelligent e-learning

Emotion recognition is vital for understanding the influence of emotions on peer interactions, academic performance, and engagement in online and virtual learning environments (Standen et al., 2020; He et al., 2022; Dehbozorgi and Kunuku, 2023).

Emotions play a crucial role in human interaction and decision-making processes. EEG signals provide an accessible, inexpensive, portable, and user-friendly means to identify emotions (Alarcao and Fonseca, 2017). This technology is highly valued for its real-time analysis capabilities of emotional states. The portability and ease of use of EEG devices make them particularly suitable for educational applications, offering new possibilities for enhancing communication, well-being, and decision-making at both individual and societal levels.

Transfer learning approaches, which utilize networks pretrained on other tasks, have proven highly effective in facial emotion recognition within human-computer interaction, achieving an impressive average accuracy of 96% (Chowdary et al., 2023). This method leverages the existing knowledge embedded in neural network models to enhance the detection of emotional expressions in digital settings. The high accuracy of these approaches lays a strong foundation for developing advanced human-computer interaction systems, which can enhance online learning experiences by providing more accurate and nuanced emotional feedback.

Chao et al. (2019) introduced a deep learning framework that employs a multiband feature matrix and a CapsNet model to improve emotion recognition from multi-channel EEG signals, outperforming common models. This innovation underscores the importance of advancing deep learning techniques to increase the accuracy and efficiency of emotion recognition in educational settings. By integrating multiple EEG channels and utilizing the generalization capabilities of CapsNet models, this framework sets a new standard for detecting emotional states, significantly impacting our understanding of emotions in academic performance and engagement in online and virtual learning environments.

The novel deep learning model (ERDL), which combines graph convolutional neural networks and LSTMs, has achieved superior classification accuracy for emotion recognition from EEG signals compared to current state-of-the-art methods (Yin et al., 2021). This advancement underscores the effectiveness of integrating various deep learning techniques to enhance emotional recognition in brain signals. By combining the capability to model complex relationships in graph-like data with the ability to handle temporal sequences through LSTMs, the ERDL model emerges as a potent tool for deciphering emotions via EEG signals. This improvement in classification accuracy is crucial for designing more effective educational interventions tailored to the emotional needs of students.

Development began in 2014 of a technique using convolutional neural networks that effectively learns emotion-relevant features from speech, maintaining stable and robust performance even in complex environments (Mao et al., 2014). This study demonstrates the power of convolutional neural networks in extracting distinct emotion-related features from speech, enabling precise and reliable recognition of emotional expressions across various settings. The consistent and robust performance of this method supports its potential for practical applications, including enhancing human-computer interaction in virtual and online educational settings.

Furthermore, research indicates that students’ understanding of emotions correlates positively with their academic performance, peer acceptance, and school adaptation, especially among children from middle-class families (Voltmer and von Salisch, 2017). This finding highlights the importance of emotional intelligence in the educational and social contexts of students, influencing various aspects of their development. The ability to understand and manage emotions not only affects academic success but also enhances the quality of interpersonal relationships and adaptability in school settings. Additionally, the variation in these associations across different socioeconomic backgrounds emphasizes the need for equitable attention to emotional development within education.

#### 3.2.2 Emotionally intelligent e-learning systems and adaptive learning systems

Emotionally Intelligent E-learning Systems (EIES) and adaptive learning systems are transforming learning experiences by providing personalized educational environments (Ninaus et al., 2019; He et al., 2022; Dehbozorgi and Kunuku, 2023).

The Emotionally Intelligent E-Learning System (EIES), based on the Bayesian Network model, accurately predicts students’ emotions during online learning sessions, enhancing the quality of virtual education (Daouas and Lejmi, 2018). This innovation underscores the importance of incorporating emotional intelligence into online learning environments. By predicting emotions, EIES can dynamically tailor the delivery of educational content, provide personalized feedback, and offer emotional support resources when necessary. This capability significantly enriches the online learning experience, creating a more responsive and engaging educational environment.

Additionally, it has been demonstrated that artificial intelligence techniques can enhance adaptive e-learning platforms by creating detailed learner profiles and models, which in turn improve the learning process and reduce uncertainty (Colchester et al., 2017). This advancement highlights the crucial role of artificial intelligence in personalizing online education by enabling systems to adapt dynamically to individual learner needs. Advanced algorithms help these platforms identify specific learning patterns, preferences, and challenges of each student, thereby facilitating the delivery of relevant and effective educational content. This adaptive capability significantly improves the learning experience, fostering a more responsive and student-centered educational environment.

A cloud-based adaptive learning system has proven effective in integrating mobile devices into the classroom environment, providing real-time feedback and context-aware content adaptation, leading to significant improvements in student performance and achievement (Nedungadi and Raman, 2012).

This approach demonstrates the potential of mobile technology and cloud computing to enhance the classroom learning experience by offering greater flexibility and personalization of educational content. By leveraging mobile devices like tablets or smartphones, adaptive systems can deliver instant feedback and tailor content to individual needs and learning contexts, thus boosting the overall effectiveness of the educational process and encouraging student participation and engagement.

Adaptive learning technologies, which tailor instruction to align with students’ personal interests, have demonstrated the potential to enhance performance and learning outcomes (Walkington, 2013). This finding emphasizes the importance of customizing educational content to match individual preferences to optimize the learning process. Adaptive algorithms analyze students’ learning patterns and interests, allowing systems to present relevant content and challenges that maintain their motivation and engagement. This personalized approach promotes more active participation and a deeper understanding of the material, ultimately leading to improved academic performance and more positive learning outcomes.

Personalized adaptive learning, facilitated by intelligent learning environments, combines personalized and adaptive learning strategies, making adaptive adjustments to teaching approaches based on individual characteristics, performance, and personal development (Peng et al., 2019). This integration of educational methods offers a comprehensive and effective solution tailored to the unique needs of each learner. By merging personalized educational content with dynamic adaptations in teaching methodology, it creates an educational environment that continually adjusts to the abilities, interests, and preferences of students. This not only maximizes each individual’s learning potential but also enhances engagement and motivation toward the educational process.

In summary, the integration of Emotionally Intelligent E-Learning Systems (EIES) and adaptive learning systems significantly enhances the educational experience by providing personalized environments that dynamically adapt to the emotional and learning needs of students. This synergy between advanced technologies and contemporary educational methodologies supports the accurate prediction of students’ emotions during online learning sessions and the real-time adaptation of content and teaching strategies. Collectively, these advancements underscore the transformative role of technology in education, promoting more effective, inclusive, and student-centered learning environments.

#### 3.2.3 Positive emotional states and academic performance

Positive emotional states correlate strongly with improved academic performance and increased engagement in online learning environments. Students’ expressed emotions, such as relief, satisfaction, and frustration, are directly linked to their academic outcomes, illustrating the significant impact of emotions on learning results (Ninaus et al., 2019; He et al., 2022; Dehbozorgi and Kunuku, 2023).

Academic emotions, ranging from anxiety to other emotional states, have a significant impact—both positive and negative—on students’ motivation, learning strategies, self-regulation, and academic performance (Pekrun et al., 2017). This study illustrates how different emotional states can affect various aspects of academic performance and student engagement. Anxiety, for instance, can impede motivation and self-regulation, while positive emotions can enhance learning strategies and promote greater engagement with study materials. Understanding the interaction between emotions and academic performance underscores the importance of creating an educational environment that promotes positive emotional states and provides support to effectively manage negative emotions.

Moreover, students’ emotions, whether negative or positive, significantly influence their academic performance, with cognitive processes and effortful control playing a moderating role in this relationship (Valiente et al., 2012). This study highlights the complex interplay between emotions and cognitive processes in the educational context, noting how effortful control can modulate the impact of emotions on academic performance. Positive emotions can enhance performance by promoting greater motivation and engagement, while negative emotions may hinder performance by interfering with attention and memory. The role of effortful control suggests that emotional and cognitive regulation strategies can mitigate the negative effects of adverse emotions and amplify the benefits of positive emotions on academic performance.

Positive emotions, such as enjoyment and pride, are positively associated with mathematics achievement, while negative emotions, such as anger, anxiety, shame, boredom, and hopelessness, have a negative correlation (Pekrun et al., 2017). This emphasizes the importance of emotions in the academic context and their differential impact on student performance. Positive emotions can boost motivation and readiness for learning, whereas negative emotions can generate distractions and cognitive blocks. These findings highlight the need to foster an educational environment that encourages positive emotions and provides effective strategies to manage negative emotions, aiming to improve both academic performance and student well-being.

Positive emotions also promote academic performance in college students when mediated by self-regulated learning and motivation (Mega et al., 2014). This study demonstrates that positive emotions not only directly influence academic performance but also interact with internal processes such as self-regulation of learning and motivation to enhance educational outcomes. Positive emotions can increase perseverance, attention, and the effectiveness of self-regulated learning strategies, improving comprehension and retention of academic material. Additionally, these emotions can reinforce intrinsic motivation and disposition toward learning, leading to deeper and more sustained engagement with the educational process.

Lastly, positive emotions positively influence problem-solving patterns by engaging students in self-regulatory activities, whereas negative emotions result in less variety of search activities and fewer regulatory activities (Zhou, 2013). This study shows how emotions can shape the way students approach academic challenges and handle complex problems. Positive emotions encourage active exploration, creativity, and cognitive flexibility, leading to a wide range of problem-solving strategies and greater solution-finding effectiveness. Conversely, negative emotions can restrict students’ ability to think creatively and seek alternative solutions, resulting in less diversity in problem-solving strategies and approaches. These findings underscore the importance of promoting a positive emotional classroom environment to foster the development of effective problem-solving skills and self-regulation in students.

Positive emotions are associated with higher academic performance as they enhance psychological capital, which includes elements like efficacy, hope, optimism, and resilience (Carmona-Halty et al., 2019). This association supports the idea that positive emotional states correlate with more effective cognitive processes, better academic outcomes, and greater engagement in online learning environments.

The emotional climate of the classroom also has a significant impact on academic achievement, fostering greater student participation across all grade levels and genders (Reyes et al., 2012). This supports the view that a positive emotional environment in the classroom is crucial for academic success as it enhances student engagement and involvement in the educational process, thereby improving learning outcomes.

From a broader perspective, the TPACK framework emphasizes the effective integration of technological, pedagogical, and disciplinary content to enhance learning outcomes (Alemán-Saravia and Deroncele-Acosta, 2021). In this context, attention-based convolutional recurrent neural networks (ACRNN) are notable for their ability to accurately extract discriminative features from EEG signals, improving emotion recognition over other methods (Tao et al., 2020). The integration of AI into educational design influences learning outcomes directly, increasing motivation, self-efficacy, and the effectiveness of cognitive learning strategies within learning communities (Stefanou and Salisbury-Glennon, 2002). Moreover, AI’s role in detecting students’ emotions not only enhances productivity and academic performance (AlZu’bi et al., 2022) but also streamlines teaching practices by allowing educators to monitor emotional states and provide targeted feedback that positively affects learning outcomes (Deniz et al., 2019).

Additionally, AI can automate assessment-related decisions, optimizing the effectiveness of computerized formative assessments to enhance student learning (Shin et al., 2022) and predict student performance with high accuracy, enabling early interventions and ensuring equitable quality education (Jokhan et al., 2022). AI applications in education are diverse, including profiling, assessment, adaptive systems, personalization, and intelligent tutoring systems (Zawacki-Richter et al., 2019).

Furthermore, course-related discussions and interactions among students are shown to positively influence grades more than non-course-related topics, underscoring the importance of emotional engagement in learning.

Scientific evidence indicates that discussions within online learning management systems can enhance student engagement, improve the content and quality of work, and lead to better learning outcomes (King et al., 2021). Aligning with this, another study suggests that highly interactive online courses—marked by substantial student-to-student and student-to-instructor interactions—are perceived more favorably in terms of engagement and learning outcomes compared to less interactive group courses and discussions (Tsai et al., 2021). Additionally, peer discussions have been shown to enhance student performance on conceptual questions in class, fostering greater understanding and improved accuracy, even when none of the students initially know the correct answer (Smith et al., 2009).

Therefore, classroom interaction and discussion are crucial factors for learning, and promoting these should be a priority within educational systems. The integration of AI can support this goal, as AI techniques can effectively identify significant contributions and patterns in students’ electronic discussions. This capability assists teachers in fostering productive discussions and enhancing learning (McLaren et al., 2010). Furthermore, AI can also be utilized to develop students’ skills in complex interpersonal behaviors, such as effective listening, teamwork, and communication (Hoffmann-Longtin et al., 2018).

### 3.3 Special education and assistive technology

Information and communication technologies (ICT) and assistive technologies are vital for helping students, both with and without disabilities, to recognize their emotions and enhance their learning. These technologies are particularly crucial in removing barriers for children with learning difficulties. Research shows that ICT applications can create inclusive learning environments and provide essential support for students with learning challenges (Standen et al., 2020; Begum et al., 2023).

Educators can utilize ICT and assistive technologies to customize learning experiences based on the emotional needs of individual students, thereby improving their engagement and overall learning outcomes. The incorporation of these technologies not only aids in emotion recognition but also establishes a supportive learning environment that promotes both emotional well-being and academic success for students with diverse learning needs (Standen et al., 2020; Begum et al., 2023).

For students with special needs, mobile learning provides greater accessibility and richer learning experiences, presenting a valuable alternative to traditional assistive devices. This mode of learning enables students with diverse needs, including those with disabilities, to engage in more adaptive and personalized learning (Standen et al., 2020).

By leveraging mobile technologies, educators can create inclusive learning environments that cater to individual learning styles and preferences, thereby enhancing student engagement and academic outcomes. Mobile devices are portable and versatile, making learning more accessible and convenient for students with special needs, allowing them to interact with educational content in a manner that best suits their specific requirements.

The integration of mobile learning not only improves accessibility but also enables students with special needs to participate more actively in their learning process, fostering independence and encouraging self-directed learning (Standen et al., 2020).

Overall, the strategic use of ICT and assistive technologies not only facilitates the identification of emotions but also nurtures an environment conducive to learning, supporting the emotional well-being and academic achievements of students with diverse learning needs.

### 3.4 Affective computing

Affective computing is an interdisciplinary field that develops systems capable of recognizing, interpreting, and responding to human emotions.

This field of research has significant relevance in education because emotions are critical to the learning process and to creating meaningful educational experiences.

Understanding students’ emotions allows educators to tailor their teaching methods to more effectively meet individual needs and foster a positive and stimulating learning environment. In the context of our research on integrating artificial intelligence to assess emotions in learning environments, we investigate how advancements in affective computing can enhance the assessment of students’ emotional experiences and improve learning outcomes.

#### 3.4.1 Affective computing as an area of AI in emotional management

As explained by Villegas-Ch et al. (2023), affective computing is a branch of Artificial Intelligence (AI) developed to enable computer systems to interact with humans effectively. This interaction is facilitated through computer vision techniques and machine learning algorithms. The primary goal is to produce a system that can elicit effective responses from users. Affective computing is interdisciplinary and consists of four main research areas: (1) Analysis and characterization of affective states, (2) Automatic recognition of affective states through facial expressions, linguistic features, posture, gaze tracking, and heart rate, among others, (3) System adaptation to respond appropriately to the users’ affective states, and (4) Design of avatars that display suitable affective responses for better user interaction.

Emotion recognition via facial expressions, often referred to as facial expression recognition, is a widely addressed topic within the field of affective computing (González-Hernández et al., 2018). By recognizing facial expressions, educators can offer more personalized responses, provide emotional support when needed, and promote a more empathetic and student-centered learning environment. Thus, the ability to recognize emotions through facial expressions is essential for enhancing the quality and effectiveness of education.

#### 3.4.2 Optimizing learning through affective computing

The assertion that affective computing is essential to intelligent learning systems is strongly supported by the growing recognition that emotions are integral to cognitive and decision-making processes. Research such as Shobana and Kumar (2021) demonstrates that emotions significantly influence perception and learning. Integrating affective computing into educational systems enables a more precise and personalized response to students’ emotional needs, thereby enhancing the effectiveness of the teaching and learning process. Recognizing and responding to students’ emotions opens new possibilities for creating empathetic and effective learning environments, ultimately fostering deeper and more meaningful learning experiences.

One of the key challenges of affective computing is the automatic detection and classification of users’ emotional reactions to learning materials (Ninaus et al., 2019). This capability is crucial in education for several reasons. Firstly, it allows educational systems to adapt personally to the emotional needs of students, enhancing the learning experience and fostering a more responsive and empathetic environment. Additionally, it enables the early identification of potential emotional difficulties that may impact academic performance, allowing for timely educational interventions.

There are online learning platforms that utilize affective computing principles to accurately identify six fundamental emotions: happiness, disgust, anger, surprise, sadness, and fear (Aly et al., 2023). Recognizing and addressing this range of emotions allows educational strategies to be more contextualized and effective. For example, detecting happiness can lead to the reinforcement of student achievements, maintaining a motivating environment. Recognizing disgust can help avoid content that triggers negative reactions, thus enhancing the learning experience. By identifying anger, platforms can provide additional support to help students overcome challenges and stay motivated. Surprise can indicate moments of insight, which can be leveraged to deepen understanding. Recognizing sadness is essential for providing emotional support, while identifying fear can signal the need for psychoeducational interventions to manage stress and ensure effective learning. Overall, these capabilities facilitate a more adaptive and emotionally-aware approach, promoting a more inclusive and effective educational environment.

Research has extensively explored methods and models for affect detection systems capable of analyzing conventional modalities such as facial expression, voice, body language and posture, physiology, brain imaging, and multimodal systems. This research connects human emotions to learning, organizing them into four quadrants—curiosity, confusion, frustration, hope—with emotions on the horizontal axis and learning on the vertical axis (Kort et al., 2001).

Affective computing offers multiple benefits when integrated with artificial intelligence for emotion recognition. It has been shown to enhance e-learning applications by detecting and responding to the emotions of learners, potentially improving the learning process (Thompson and McGill, 2012). It can adjust the mood of learners to create a more effective learning environment (Chen and Lee, 2012), recognize emotions from speech using neural networks (Zhang et al., 2007), monitor students’ behavior to gauge their attention and engagement levels, and support effective learning processes (Bevilacqua et al., 2009). Additionally, it can boost motivation and satisfaction in game-based adaptive learning systems (Tsai et al., 2012). In the context of game-based learning, one study shows that adaptive gamification—which combines artificial intelligence, gamification, and educational data mining—has a positive impact on student engagement and learning performance (Daghestani et al., 2020).

#### 3.4.3 Advantages of affective computing as an intelligent educational system

The primary aim of affective computing is to develop an “intelligent” computer system capable of sensing, recognizing, understanding, and intelligently responding to human emotions in a timely and friendly manner (He et al., 2022). Affective computing is an interdisciplinary field dedicated to creating systems and technologies that can recognize, interpret, process, and respond to human emotions. This field strives to equip machines with the capability to comprehend and mimic human emotional intelligence, utilizing a variety of data sources like facial expressions, tone of voice, handwriting patterns, and other physiological indicators to ascertain a person’s emotional state.

In educational settings, when integrated with artificial intelligence, affective computing can personalize teaching by adapting content to align with students’ emotions and individual needs (Kratzwald et al., 2018; Marín-Morales et al., 2018, 2020; Arnau-González et al., 2021; Li S. et al., 2021; Li W.-C. et al., 2021; Wang et al., 2022). As such, affective computing is a crucial element in the application of artificial intelligence in emotional management within educational environments. It allows AI to interpret facial expressions, voice tones, and other emotional cues, providing insights into students’ emotional states. This capability not only facilitates the personalized adaptation of educational content but also enables the early identification of potential emotional challenges that may impact academic performance. Overall, the integration of affective computing into educational emotional management not only enhances the effectiveness of learning environments but also supports the emotional well-being of students, fostering a more supportive and responsive educational setting.

Several advantages and contributions of affective computing are recognized in recent research. For example, the Probability and Integrated Learning (PIL) algorithm effectively recognizes high-level human emotions, offering potential benefits for affective computing (Jiang et al., 2020). Additionally, fuzzy cognitive maps can accurately predict artificial emotions, aiding in the design of affective decision-making systems within AI (Salmeron, 2012).

In the context of e-learning systems, affective computing involves using tools to recognize users’ emotions and adapt educational systems accordingly (Jaques and Viccari, 2006). It has been demonstrated that affective computing can detect human attention levels using multimodal inputs such as webcams and mouse movements, potentially enhancing performance in intelligent e-learning applications (Li et al., 2016).

Broadly speaking, affective computing is a field within artificial intelligence that focuses on developing systems capable of recognizing, interpreting, processing, and simulating human emotions. It employs machine learning techniques, computer vision, natural language processing, and other disciplines to analyze and respond to the emotions expressed by users. The ultimate goal is to create systems that are more empathetic and can interact more naturally with people. Particularly in education, integrating affective computing is crucial for understanding and addressing student emotions, promoting a learning environment that is more personalized, effective, and attentive to emotional well-being.

## 4 Conclusion

The integration of assistive technology, information and communication technology (ICT), and artificial intelligence (AI) in educational settings has revolutionized the support available to students, particularly those with learning difficulties, in managing their learning and emotions. For children with diverse learning needs, AI-enhanced emotion detection, personalized learning experiences through ICT, and improved accessibility via assistive technology have significantly reduced learning barriers.

This research highlights the critical role of technology in enhancing emotion recognition, creating inclusive learning environments, and promoting academic success for all children. By employing these advanced tools, educators can develop customized learning plans, provide immediate feedback, and support both the academic and emotional development of students with and without disabilities.

This comprehensive approach to integrating AI, ICT, and assistive technology not only enhances emotional support but also equips students with the tools they need to actively participate in their education. Ultimately, this opens the door to a more successful and inclusive educational process.

### 4.1 Limitations

This study aimed to review and analyze the existing literature on the integration of artificial intelligence for evaluating emotions in educational environments. The review relied on articles sourced from specific academic databases, including PubMed, Web of Science, PsycINFO, and Scopus. While these databases are significant in the scientific community, it is crucial to note that this selection might have limited the inclusion of pertinent research published in other sources or in gray literature.

Additionally, a linguistic bias is acknowledged; the review covered articles in English, Spanish, and Portuguese, but research published in other languages was not considered. This restriction might have excluded studies that could provide valuable insights, affecting the geographical and cultural representativeness of the studies included in this analysis. Consequently, the generalizability of the findings to different educational and cultural contexts may be limited.

Lastly, we recognize an open access bias. Despite efforts to include a diverse range of academic sources and databases, some studies may be behind paywalls. This limitation could have excluded significant research, impacting the comprehensiveness of the review. Access was restricted to studies that were either open access or available through institutions with subscriptions. Therefore, caution is advised when interpreting the findings of this review, as they may not comprehensively reflect all the available research in the field of AI-driven emotional assessment in education.

#### 4.1.1 Temporal limitation

The review findings may not fully represent the latest advances or developments in AI-driven emotional assessment within education, as the search was conducted up to the year 2023. Consequently, emerging technologies, methodologies, or ethical considerations may not be sufficiently covered, potentially limiting the relevance and applicability of the study’s findings to current educational practices.

#### 4.1.2 In terms of generalizability

While the review offers insights into the integration of AI for emotional assessment in educational settings, the findings may not be broadly applicable across diverse educational environments, student populations, and cultural contexts. Differences in educational infrastructure, resources, and practices among various regions or institutions could influence the feasibility and effectiveness of implementing AI-driven approaches to emotional assessment.

### 4.2 Ethical considerations

Although the study acknowledges the importance of ethical considerations in the development and implementation of AI technologies within education, the review itself does not delve into the ethical implications of using AI for emotional assessment. Further exploration of ethical frameworks, privacy concerns, and potential social impacts is needed to ensure responsible and equitable implementation of AI technologies within educational settings.

## Data availability statement

The original contributions presented in the study are included in the article/Supplementary material, further inquiries can be directed to the corresponding author.

## Author contributions

AV: Writing – review & editing, Writing – original draft, Visualization, Validation, Supervision, Methodology, Investigation, Formal analysis, Data curation, Conceptualization. AD-A: Writing – original draft, Methodology, Formal Analysis, Data curation, Validation, Investigation, Writing – review & editing, Visualization, Conceptualization. JA: Writing – review & editing, Visualization, Validation, Supervision, Resources, Investigation, Conceptualization. AB: Supervision, Project administration, Writing – review & editing, Visualization, Methodology, Conceptualization. CL-G: Validation, Methodology, Writing – review & editing, Visualization, Supervision, Conceptualization. MM-G: Writing – review & editing, Visualization, Supervision, Resources, Project administration, Investigation, Conceptualization.
