# Supplementary material for: Integrating artificial intelligence to assess emotions in learning environments: a systematic literature review
Source: Front Psychol. 2024 Jun 19;15:1387089. doi: 10.3389/fpsyg.2024.1387089 (PMC11223560; doi:10.3389/fpsyg.2024.1387089)
Supplement: Supplementary file 1 [file Table_1.DOCX]

**Table 1.**

|  | **Objectives** | **Method and sample** | **Results** |
| --- | --- | --- | --- |
| **Country**: Colombia  **Year of Publication**: 2002  (Conati, 2002) | To present a probabilistic model that monitors a user's emotions and engagement during interactions with educational games.  The model aims to assess affect by integrating evidence on both the possible causes of the user's emotional arousal (state of the interaction) and its effects (bodily expressions influenced by emotional reactions). | Users or students engaging with educational games in the theoretical framework of the model presented in the article.  The probabilistic model relies on a Dynamic Decision Network to leverage any indirect evidence on the user's emotional state, to estimate this state and any other related variable in the model. | The article presents a probabilistic model for monitoring a user's emotions and engagement during interactions with educational games.  The model integrates evidence on both the possible causes of the user's emotional arousal (state of the interaction) and its effects (bodily expressions influenced by emotional reactions).  Implemented using a Dynamic Decision Network, the model aims to help decision-theoretic pedagogical agents generate interventions that balance a user's learning and engagement effectively during educational game interactions.  The focus is on assessing affect by leveraging any available information on the user's emotional state while explicitly expressing uncertainty in predictions when information is limited or ambiguous.  The model is designed to address the challenge of recognizing a variety of emotional states during interactions, rather than constraining the task or granularity of emotion recognition.  The article emphasizes the importance of detecting specific emotions in educational games to tailor interactions based on the user's emotional state and enhance the effectiveness of computer-based educational games. |
| **Country**: China  **Year of Publication**: 2012  **Study date**:  (Qi, 2012) | To address emotion deficiency in the current E-Learning systems by developing a system that can accurately recognize and classify emotions in speech.  This system aims to provide emotion encouragement and compensation based on the specific emotion state detected in the learner's speech. | Experimental subjects were provided with a list of emotional sentences and directed to express their emotions naturally by recalling emotional experiences from their lives. Subjects from different language backgrounds were selected to ensure the development of a language-independent system. The speech utterances were recorded in English and Chinese, with over 500 utterances for each of the six emotional classes: happiness, sadness, anger, fear, surprise, and disgust. | The system employed Sequential Forward Selection (SFS) with a General Regression Neural Network (GRNN) along with a consistency-based selection method to identify and extract potential prosodic features from emotional speech data.  Using a standard neural network (NN) with 17 features, the system achieved an overall correct recognition rate of 77.24%. With 12 selected features, the recognition rate improved to 80.69%.  The K-nearest Neighbors classifier (KNN) on the 12 selected features achieved an overall recognition rate of 79.31%.  The proposed Modular Neural Network (MNN) with the 12 selected features outperformed other classifiers, achieving the best overall classification accuracy of 83.31%.  The system demonstrated the ability to identify emotional expressions in speech beyond language boundaries, emphasizing the universal nature of emotion and its vocal expresión. |
| **Country**: United States  **Year of Publication**: 2017  (Stepan K et al., 2017) | to assess the impact of using VR technology compared to traditional online textbooks in enhancing students' understanding of neuroanatomical structures. | A randomized controlled study with 66 medical students (33 in both the control and experimental groups). | There was no significant difference in anatomy knowledge between the 2 groups on preintervention, postintervention, or retention quizzes. The VR group found the learning experience to be significantly more engaging, enjoyable, and useful (all p < 0.01) and scored significantly higher on the motivation assessment (p < 0.01).  Immersive VR educational tools awarded a more positive learner experience and enhanced student motivation. However, the technology was equally as effective as the traditional text books in teaching neuroanatomy. |
| **Country**: China  **Year of Publication**: 2019  (Liu & Wu, 2019) | To introduce an affective arousal evaluation system that utilizes artificial intelligence techniques to assess emotional responses during the teaching process. | The system embeds facial expression recognition and speech emotion recognition methods, which extract and analyze the video streaming collected by the ubiquitous web cameras from both teacher side and student side during the online teaching process. The experiment verifies the correlation and Granger causality between teacher-student affective sequences. The implementation of the system realizes the output of three quantitative indicators: “Affective Frequency Index”, “Affective Correlation Index” and “Affective Arousal Level”. | The system uses the face++ expression recognition algorithm, achieving a 7-category emotion recognition accuracy of 73.95% and a triple classification accuracy of 84.13% when tested on the Extended Cohn-Kanade facial expression database.  The Mel Frequency Cepstral Coefficient (MFCC) based speech recognition algorithm, tested on the CASIA Chinese emotional speech corpus, reached an accuracy of 80.4% 1.  The prototype system aims to monitor emotional interactions between teachers and students in online classes, providing automatic teaching quality assessment based on affective evaluation. It consists of three layers: input layer, data processing layer, and output layer.  The system outputs three quantitative indicators: "Affective Frequency Index," "Affective Correlation Index," and "Affective Arousal Level" based on emotion-related scored time series.  Comparative experiments showed consistency between manually labeled emotional sequences and systematically extracted ones for teachers and students. The system output also demonstrated consistency in emotional sequences, indicating the system's effectiveness in evaluating affective arousal in education. |
| **Country**: Germany  **Year of Publication**: 2019  (Ninaus et al., 2019) | We employed both automatic facial emotion detection as well as subjective ratings to evaluate emotional engagement of adult participants completing either a game-based numerical task or a non-game-based equivalent. | 150 adult university students. took part in the study after having given their written informed consent (age M = 23.69 years, SD = 4.25). | Using a machine learning approach on facial emotion detection data we were able to predict whether individual participants were engaged in the game-based or non-game-based task with classification accuracy significantly above chance level. Moreover, facial emotion detection as well as subjective ratings consistently indicated increased positive as well as negative emotions during game-based learning. These results substantiate that the emotionally engaging nature of games facilitates learning. |
| **Country**: Mexico  **Year of Publication**: 2018  (González-Hernández et al., 2018) | This work presents the application of a convolutional neural network (CNN) used to identify emotions through taken images to students, which are learning Java language with an Intelligent Learning Environment. | The databases were built using 10 professional French-speaking theater actors who were trained by a professional director. The corpus is comprised of over 7000 audiovisual emotion representations. Facial expression databases. | The results show a comparison among three emotion recognition systems. One applying a local binary pattern approach with facial patches, another applying a geometry-based method, and the last one applying the convolutional network. The analysis presented satisfactory results; the CNN obtained a 95% accuracy for the RaFD database, an 88% accuracy for a learning-centered emotion database and a 74% accuracy for a second learning-centered emotion database. Results are compared against the classifiers support vector machine, k-nearest neighbors, and artificial neural network. |
| **Country**: Tunisia.  **Year of Publication**: 2018.  (Daouas & Lejmi 2018). | The aim is to enhance the quality of Elearning processes by recognizing and responding to students' emotions, ultimately improving the overall learning experience. | The sample consisted of 101 participants, including 79 subjects in Sample 1 (30 women and 49 men, computer engineers and workers in multinational companies) and 22 subjects in Sample 2 (12 women and 10 men, students in various fields). The participants were asked to contribute to the experimentation by filling out questionnaires to determine their personality traits, goals, and emotions for different events during Elearning sessions. The data collected from the participants were organized into training and testing sets for machine learning algorithms. | Development of a computational probabilistic model of emotions using a Bayesian Network (BN) model to predict students' emotional states during Elearning sessions.  Implementation of a simulation of Emotionally Intelligent Elearning System (EIES) based on the BN model, which was able to accurately predict students' emotions when events occurred in the Elearning interface.  Validation test results showed that the EIES system predicted emotional states correctly in 83.75% of cases, demonstrating the effectiveness of incorporating emotional intelligence into Elearning environments.  The collected data from questionnaires filled out by participants were used to train and test the BN model, with 711 cases in the training set and 198 cases in the testing set.  The study highlighted the importance of considering emotional intelligence in Elearning systems to enhance the quality of distant learning experiences. |
| **Country**: United States  **Year of Publication**: 2018  (Kim et al., 2018) | to propose a new system design paradigm for smart classrooms that integrates real-time sensing and machine intelligence to create emotionally aware learning environments.  The paper aims to address current issues in traditional classrooms, such as lack of personalized feedback and limited interaction, by leveraging AI technologies to enhance communication and learning outcomes in educational settings. | The proposed system is capable of making real-time suggestions to a presenter in class to improve the quality and memorability of their presentation by enabling the presenter to make real-time adjustments/corrections to their non-verbal behavior, such as hand gestures, facial expressions, and body language. We base our suggested system components on existing research in affect detection, deep learning-based emotion recognition, and real-time mobile cloud computing. We provide a comprehensive study of these technologies and determine the computational requirements of a system incorporating these technologies. Based on these requirements, we provide a feasibility study of the system. | The system captures nonverbal cues such as facial expressions, body movements, speech prosody, and pupil dilation to evaluate the needs and capabilities of individual students.  Researchers have been developing intelligent tutoring systems for individualizing instruction, strategy suggestions, and feedback to students based on their performance and learning styles.  In the Training Mode, the system records presenter's behavioral cues and listeners' ratings to train the machine intelligence system, with the crowd evaluating the presentation based on various factors like body language and affect.  The system provides real-time feedback to presenters during their presentations through haptic gloves or emojis displayed on a screen, aiming to improve the scores received from the audience.  The selection criteria for questions used in evaluation are based on educational theories, provide meaningful psychometrics, offer monotonically increasing quantitative answers, and are statistically independent to avoid cognitive overload on the crowd.  The design of the Crowd Annotation Engine involves collecting raw human ratings from experts and peers through a smartphone app, quantifying these ratings into metrics, and integrating them into the machine learning algorithm to evaluate the presenter's voice, affect, and body language. |
| **Country**: India  **Year of Publication**: 2019  (Sharma & Mansotra, 2019) | To implement a multimodal sentiment prediction system that can analyze the emotions predicted from different modal sources such as video, audio and text and integrate them to recognize the group emotions of the students in a classroom. | In this study, the authors used the Facial Emotion Research (FER) database. This database has 48x48 pixel size images of faces and it can predict seven different emotions such as anger, fear, sad, happy, neutral, disgust and surprise. Each image in this database is labelled with appropriate emotions as a numerical code ranging from 0-6. | The implemented system framework was tested in a classroom environment during a live lecture and the predicted emotions demonstrated the classification accuracy of our approach. As discussed in the earlier sections, Haar cascades based facial features extraction was very helpful for emotion classification.  We had selected the transfer learning approach in CNN for the classification of facial emotions, primarily, due to its ability to reduce the time spend in training the model, especially with the large FER2013 dataset. This in turn resulted in improved predictions and classification accuracy. For the classification of sentiments using speech, we have combined the RNN approach with LSTM which can be more efficient when working with large and non-linear datasets. The twitter text-based classification of sentiments was done using a hybrid approach with lexicon and SVM based feature learning that performed exceptionally well. Our decision to use decision-level fusing of the features worked well in integrating the sentiments from the different modal schemes into one valid feature representing the classified sentiments. |
| **Country**: Morocco.  **Year of Publication**: 2019.  (Ouherrou et al, 2019) | To explore the benefits of ICT use to identify the ways in which emotions are involved during the learning process in Virtual Learning Environments (VLE). | A total of 42 children (native Arabic speakers) participated in this study and they were  Divided into two groups, control and experimental group. In this study, they  used an educational game, that supports  children with dyslexia in reading and writing in Arabic. They used advances in Artificial  Intelligence (AI) to detect children’s emotions through their facial expressions  by analyzing seven basic facial emotion expressions (angry, disgust, fear, happy,  sad, surprise and neutral) while playing an educational game. | The initial results indicate that emotions are present in Virtual Learning Environments *(*VLE) and they appear to suggest that children with LDs experience the same emotions as their peers without LDs in VLE.  Besides, they show that children with LDs experience less negative emotions compared to literature evidence about the presence of a higher level of negative emotions in classroom.  Learners might experience different types of emotions in VLE. According to the diversity of the target children’s emotional problems, it is important to ensure ICT applications that provide the appropriate learning environment to motivate and give them the psychological support. |
| **Country**: England.  **Year of Publication**: 2020.  (Standen et al, 2020) | The study aims to evaluate how this innovative technology can support personalized learning experiences for learners with intellectual disabilities and improve their engagement, affective states, and achievement levels. | The article employed a research methodology that involved the development and evaluation of an adaptive learning system based on multimodal affect recognition for learners with intellectual disabilities. The study utilized machine learning algorithms to automatically detect affective states, such as boredom, frustration, and engagement, during learning sessions.  Data collection involved the participation of teachers, parents, and students with intellectual disabilities who provided consent for the study. The research team analyzed the data to assess the impact of the adaptive learning system on learning achievement and emotional states of the participants. The study also compared sessions using the adaptive learning system with and without affect sensing to evaluate the system's effectiveness in enhancing engagement and reducing boredom.  A sample of 67 participants aged between 6 and 18 years with intellectual disabilities took part in the study. The participants acted as their own controls in a series of learning sessions using the adaptive learning system based on multimodal affect recognition. The sessions alternated between using the system with both affect detection and learning achievement to drive the selection of learning content (intervention) and using learning achievement alone to drive the selection of learning content (control). Data from these sessions were analyzed to assess the impact of the system on engagement, frustration, boredom, and learning achievement of the participants. | The results of the study on the adaptive learning system based multimodal affect recognition for learners with intellectual disabilities indicated several key findings:   1. Lack of boredom was strongly linked to learning achievement, with both frustration and engagement positively related to achievement. 2. Sessions using the adaptive learning system with affect sensing showed higher levels of engagement and lower levels of boredom compared to sessions without affect sensing. 3. While there was a significant increase in engagement and decrease in boredom in intervention sessions, there was no significant difference in learning achievement between intervention and control sessions. 4. The study highlighted the importance of tailoring learning activities to the emotional states and needs of learners to enhance engagement and promote positive affective states that support learning.   These results suggest that adaptive learning systems based on affect recognition have the potential to improve the learning experiences of individuals with intellectual disabilities by personalizing learning content based on their affective states. |
| **Country**: Iran.  **Year of Publication**: 2020.  (Allahyari, 2020) | to utilize artificial neural networks to predict emotional intelligence (EI) levels in different occupational, educational, gender, and age groups.  The aim is to provide a model that can estimate EI levels across various demographics and serve as a basis for addressing potential deficiencies in each group | The study conducted on Iranian individuals aged 17-73 years utilized the BarOn emotional quotient inventory (EQ-i) to measure emotional intelligence (EI). Data from 901 participants were collected from various settings in three cities. The artificial neural network method was employed for data analysis using SPSS-22 software.  The neural network model was developed to predict EI based on variables such as age, gender, occupation, marital status, and education. The study focused on determining the impact of these sociological variables on EI and identifying the most effective neural network model for predicting EI. | The study included 901 participants, predominantly male, married, and residing in urban areas. The participants were from various occupational sectors with diverse educational backgrounds. The mean age of the participants was 35.37 years. The neural network analysis identified the most suitable model for predicting EI, which included a hyperbolic tangent function in the hidden layer and a sigmoid function in the output layer.  The neural network model demonstrated significant correlations in predicting EI across various dimensions, highlighting the advantages of using artificial neural networks over traditional regression models in predicting EI based on sociological variables. |
| **Country**: China  **Year of Publication**: 2021  (Yu, 2021) | Explores face emotion recognition based on deep learning neural networks in order to make the computer recognize facial emotion more accurately. | Database of human emotions.  The methodology was focused on creating a method for human emotion recognition based on deep neural networks, which includes three continuous layers, three fused layers and one fully connected layer. Then a large-scale independent training database was established, and experiments were conducted on a human emotion database. | The research results show that this method can quickly and accurately identify and verify human emotions.  In the future, this method can be used to construct an ingenious learning environment and provide technical support for improving human emotional communication and learning behavior. Compared with traditional recognition methods that need to manually define and perform appropriate feature extraction functions, the main advantage of deep neural networks is that it eliminates these defects, which require a lot of human factors to disturb, thereby reducing the uncertainty of recognition. In recent years, deep neural networks have made outstanding achievements in computer vision, natural language processing and other fields. Therefore, this article uses deep neural network-related recognition algorithms and deep neural network facial recognition algorithms to recognize human facial emotions.  Emotions can affect and control human intellectual activities, such as attention, memory and thinking ability. Accurate recognition of human emotions is the basis for establishing continuous emotional communication in an intelligent learning environment, and it is also an important way to assess human learning conditions. Traditional methods of recognizing human emotions have problems such as low recognition rate, complex algorithm and low intensity efficiency, and it is easy to lose key information about facial features. |
| **Country**: China  **Year of Publication**: 2021  (Huang & Zhang, 2021) | In view of the above problems, this study introduces the face recognition algorithm based on a student classroom video and its implementation process, improves the hybrid face detection model based on a traditional model, and proposes the neural network algorithm of student expression recognition based on a visual transformer. | A database of faces was used for the work.  The methodology followed the following steps:   1. Description of Algorithm. 2. Data Preparation. 3. Feature Extraction. 4. Model Training. 5. Classroom Behavior Status Assessment. 6. Data Acquisition and Processing. 7. Feature Detection and Head Pose Estimation. 8. Attention Judgment 9. Facial Expression Recognition. 10. Experimental Analyses | The experimental results show that the proposed algorithm based on students’ classroom videos can effectively detect students’ attention and emotional state in class.  It proves that the intelligent analysis of students’ classroom videos will help teachers and other school administrators to make teaching scientific and improve teaching quality.  Along with the continuous development of intelligent devices such as sensors, the physiological signal and other modal information will be added to the more video intelligent analysis of college students, through a variety of modal signal complement each other, will all aspects of the analysis of students’ comprehensive emotional state, to help teachers and school administrators scientific teaching, further improve the quality of teaching.  Psychological research shows that positive emotions promote cognitive activities during learning, whereas negative emotions hinder cognitive activities. |
| **Country**: India  **Year of Publication**: 2021  (Shobana & Kumar, 2021) | This paper presents the architecture, development, evaluation of the I-Quiz system, an intelligent assessment tool, which captures and analyses both the implicit and explicit non-verbal behaviour of learner and provides insights about the learner’s real knowledge acquisition level. | 500 undergraduate engineering students.  The I-Quiz system uses an innovative way of analyzing the learner's non-verbal behavior and trains the agent using machine learning techniques. The system's intelligent agent evaluates and predicts the students' actual level of knowledge acquisition. A total of 500 engineering students were asked to attend an on-screen MCQ assessment test using the I-Quiz system comprising 20 multiple-choice questions related to advanced programming in C. The student's non-verbal behavior is recorded using a front-facing camera during the entire assessment period. The resulting dataset of nonverbal behavior and question and answer scores is used to train the random forest classifier model to predict the learner's actual level of knowledge acquisition. | The I-Quiz system uses an innovative way to analyse the learner non-verbal behaviour and trains the agent using machine learning techniques. The intelligent agent in the system evaluates and predicts the real knowledge acquisition level of learners.  The resultant dataset of non-verbal behaviour and question-answer scores is used to train the random forest classifier model to predict the real knowledge acquisition level of the learner. The trained model after hyperparameter tuning and cross validation achieved a normalized prediction accuracy of 85.68%.  The contribution to the literature is to exhibit that the non-verbal behaviour of learners could be analyzed to estimate the real knowledge level of learners during an assessment activity. The MCQ assessment tool can in real-time assess the learner and identify the specific area in which he/she lags in knowledge acquisition (even if the learner had randomly guessed and answered the question correctly). The intelligent agent as a part of the E-learning platform can help in providing a personalized learning experience to the learners. This is possible due to the ability of the agent to assess the learners with their non-verbal behaviour in a personalized manner to provide adaptive content delivery. The system can provide timely and personalized intervention points in an intelligent elearning platform. |
| **Country**: United States  **Year of Publication**: 2021  (Glassman et al., 2021) | This study aims to assess parents’ awareness of technoference and its harms, the acceptability of AI tools for mitigating technoference, and how each of these constructs vary across sociodemographic factors. | The researchers administered a web-based survey to a nationally representative sample of parents of children aged ≤5 years. Parents’ perceptions that their own technology use had risen to potentially problematic levels in general, their perceptions of their own parenting technoference, and the degree to which they found AI tools for mitigating technoference acceptable were assessed by using adaptations of previously validated scales. Multiple regression and mediation analyses were used to assess the relationships between these scales and each of the 6 sociodemographic factors (parent age, sex, language, ethnicity, educational attainment, and family income). | Of the 305 respondents, 280 provided data that met the established standards for analysis. Parents reported that a mean of 3.03 devices (SD 2.07) interfered daily in their interactions with their child. Almost two-thirds of the parents agreed with the statements “I am worried about the impact of my mobile electronic device use on my child” and “Using a computer-assisted coach while caring for my child would help me notice more quickly when my device use is interfering with my caregiving” (187/281, 66.5% and 184/282, 65.1%, respectively). Younger age, Hispanic ethnicity, and Spanish language spoken at home were associated with increased technoference awareness. Compared to parents’ perceived technoference and sociodemographic factors, parents’ perceptions of their own problematic technology use was the factor that was most associated with the acceptance of AI tools.  Parents reported high levels of mobile device use and technoference around their youngest children. Most parents across a wide sociodemographic spectrum, especially younger parents, found the use of AI tools to help mitigate technoference during parent-child daily interaction acceptable and useful. |
| **Country**: China  **Year of Publication**: 2022  (Zhang & Srivastava, 2022) | To develop a speech emotion recognition method in educational scenes based on machine learning. | Using the kernel canonical correlation analysis in machine learning algorithm, the emotional feature samples are nonlinearly mapped to the high-level feature space, the correlation between different emotional features is analyzed, the nonlinear correlation between the two groups of variables is obtained, the two speech emotional features are integrated, and the feature samples are constructed. SVM is used to establish speech emotion recognition classifier, and genetic algorithm is used to determine the optimal parameters.  The phonetic databases used in this experiment are Berlin affective phonetic database and Chinese affective corpus of Chinese Academy of Sciences. The Berlin affective corpus was recorded by the Technical University of Berlin. There are 10 non-professional actors, 5 men and 5 women. They have anger, boredom, disgust, fear, happiness, neutrality, sadness and 10 recorded scripts. A total of 800 emotional sentences were recorded, and then 20 volunteers listened and recognized them. Among the 800 emotional sentences, some sentences are short and difficult to recognize; There are also some sentences with serious colloquialism. Therefore, the samples of emotional sentences were screened. Finally, 535 sentences were retained. The Chinese emotion corpus was recorded and provided by the human-computer speech interaction research group of the State Key Laboratory of pattern recognition, Institute of automation, Chinese Academy of Sciences. There are two male and two female professional speakers. They use six emotional states: anger, fear, happiness, sadness, surprise and neutrality. They have 50 recording scripts and finally get 1200 sound emotions. Both data sets are stored in 16000 sample rate, 16 bit quantization and wav format. | The study developed a new method based on machine learning algorithms to improve the accuracy and anti-noise performance of speech emotion recognition in educational scenes.  Kernel canonical correlation analysis was used to map emotional feature samples to high-level feature space, enabling the analysis of correlations between different emotional features and their integration for improved recognition results.  Support Vector Machine (SVM) was employed to establish a speech emotion recognition classifier, and genetic algorithms were utilized to determine optimal parameters for the SVM model.  The experimental results showed that the emotion recognition rate of the proposed method was more than 90%, with specific emotions like anger, fear, happiness, and sadness achieving recognition rates of over 95%.  The method demonstrated high anti-noise performance, with speech emotion recognition results remaining consistent with actual speech emotions under different noise conditions, including Gaussian noise, salt and pepper noise, and Gaussian filter operator standard deviation.  The proposed method outperformed existing recognition methods, achieving high emotion recognition rates and demonstrating robustness against noise interference. |
| **Country**: China  **Year of Publication**: 2022  (Dan, 2022) | The purpose of this paper is to improve the theoretical system of intelligent teacher training, build an intelligent teacher training platform, create intelligent training course resources, and establish an intelligent teacher training mechanism. | This article primarily uses research and interview methods to analyze the status quo of teacher professional development, existing problems, and research needs. | The survey results showed that 46.7% of the participants were in favor of intelligent training, but there were also 70 people who believed that the biggest difficulty of intelligent training was the lack of a relatively fixed and effective platform. Therefore, it is necessary to design a smart training platform that provides a good platform for teachers to learn. |
| **Country**: Hong Kong.  **Year of Publication**: 2021.  (Ngai, el al, 2022). | The study aims to enhance emotion recognition by integrating multiple bio-signal data sources and leveraging deep learning techniques for improved accuracy in identifying emotions based on arousal and valence dimensions. | The method employed in the article "Emotion recognition based on convolutional neural networks and heterogeneous bio-signal data sources" involves a generic framework for emotion recognition using three bio-signal data sources: face, eye, and EEG data.  Participants are invited to attend experiments where bio-signal data (eye, face, and EEG) is collected using different devices.  Participants are asked to answer the Self-Assessment Manikin (SAM) questionnaire, which represents emotions in two dimensions: arousal and valence.  A multi-branch deep convolutional neural network is developed, incorporating two channels of EEG signals, 2D/3D face data, and 2D eye data for emotion recognition.  The effectiveness of the proposed system is compared with state-of-the-art baseline systems that use three or fewer input data sources. | Extensive experiments were conducted on the modality and emotion data, the results of which showed that our system has high accuracies of 67.8% and 77.0% in valence recognition and arousal recognition, respectively. The proposed method outperformed most state-of-the-art systems that use similar but fewer modalities. Moreover, the use of facial depth has outperformed the use of facial images. The proposed method of emotion recognition has significant potential for integration into various educational applications.  The study successfully integrated three important bio-signal data sources (face, eye, and EEG signals) for multi-class emotion classification, which led to improved effectiveness and accuracy in emotion recognition.  The proposed multi-branch deep convolutional neural network, utilizing EEG signals, face data, and eye data, showed promising results in emotion recognition tasks.  Comparison with state-of-the-art baseline systems revealed the effectiveness of the proposed system in utilizing multiple modalities for emotion recognition.  The study highlighted the potential of multimodal emotion recognition systems in various applications, such as education, to understand and address students' emotional needs in real-time.  Integrating heterogeneous bio-signal data sources and employing deep learning models can enhance the performance of emotion recognition systems, offering new possibilities for real-world applications and research directions. |
| **Country**: Spain  **Year of Publication**: 2022  (Llurba et al., 2022) | This study aims to explore the possibilities of using a camera for emotion recognition (ER) with a view to the potential use of this information to improve the teaching-learning process. | The methodology followed the User Centered Design (UCD), planning and analysing data, designing the sequence, developing, testing and revising. The participants were not randomly selected and were carried out in a natural classroom context. The study is a five-week longitudinal study, which is the most convenient way to capture students’ emotions at various points in time. The first week was a test, so data was not counted for the statistical analysis. The camera was placed in two subjects, in Mathematics and Chemical classes. | We have been able to create a code capable of detecting and recognising faces, identifying emotions and converting images into data, which can be processed, analysed and stored. This has allowed us to establish the occurrence and frequency of seven emotions and the neutral emotion for each student. We know we cannot draw conclusions about the content obtained as it is a pilot study, but it is possible to glimpse the potential usefulness of having systematised emotional information made possible by ER technology together with contextual variables for the teacher, and even for other educational levels, and the benefit it would have for students. We see this pilot study as a first step in exploring the potential of emotion recognition (ER) in the classroom in our cultural context.  On the basis of the results obtained, future lines of research can be considered.  - Identify more precisely the emotions expressed by students in response to specific learning content, methodologies, teaching style, and classroom environment, among other conditions.  - Monitor teachers' emotions, due to the importance of the teacher's role in the class climate, emotional development and student outcomes.  - Provide feedback to teachers and students on recorded emotions to assess their perception and usefulness for autoregulation.  - A study with a larger sample would be necessary to analyse the combined influence of emotions, academic performance and time of day.  - The evolution of the pilot study is to display the information to the teacher in real-time. |
| **Country**: United States.  **Year of Publication**: 2022.  (Kataev, Bulysheva & Mosiaev, 2022). | To explore the application of artificial intelligence methods, particularly neural networks, in the school educational process. | Using artificial intelligence technologies, specifically artificial neural networks, to analyze video data from the built-in digital cameras of laptops to assess the psycho-physiological and psycho-emotional states of schoolchildren during the learning process. The researchers developed a neural network for assessing head turns while students perform school assignments, aiming to evaluate students' states and their ability to perceive different types of educational information. The study included experiments where participants were asked to perform specific head movements while in front of a computer with a digital camera to test the effectiveness of the proposed method. | The study demonstrates the successful application of artificial intelligence methods, specifically neural networks, in monitoring students' psycho-physiological and psycho-emotional states during educational tasks. By analyzing video data from built-in digital cameras of laptops, the researchers were able to assess students' head movements and evaluate their states while performing school assignments. The experiments conducted to test the effectiveness of the proposed method showed that the neural network accurately recorded the movements of students' heads during classes. This approach provides a new method for monitoring and evaluating students' involvement in the online educational process, offering insights into students' ability to perceive different types of educational information. Overall, the results suggest that artificial intelligence technologies can play a significant role in enhancing the educational process by providing valuable information about students' states and interactions with educational tasks. |
| **Country**: Russian.  **Year of Publication**: 2022.  (Savchenko, Savchenko & Makarov 2022) | To develop a fast and accurate technique for classifying emotions and engagement in online learning environments. The aim is to implement this technique in online learning software on laptops or mobile devices without the need for powerful GPUs. | The method employed in the article involves the use of a single efficient neural network for extracting emotional features from facial expressions in online learning environments. The network is pre-trained on face identification and fine-tuned for facial expression recognition using a robust optimization technique. This allows for the fast simultaneous prediction of students' engagement levels, individual emotions, and group-level affect. The proposed framework can be integrated into existing e-learning tools for real-time assessment of students' emotions and comprehension.  The sample used in the study includes datasets from EmotiW challenges, such as EngageWild, AFEW (Acted Facial Expression In The Wild), and VGAF (Video-level Group AFfect). These datasets are utilized to evaluate the performance of the proposed neural network in classifying emotions and detecting engagement in online learning scenarios. The experimental results on these datasets demonstrate the effectiveness of the proposed network in comparison to existing models, showcasing its potential for real-time video processing and emotion classification. | The results of the study show that the proposed neural network is effective in classifying emotions and detecting engagement in online learning environments. The network is able to extract emotional features from facial expressions in real-time, allowing for the simultaneous prediction of students' engagement levels, individual emotions, and group-level affect. The experimental results on datasets from EmotiW challenges demonstrate that the proposed network outperforms existing models in terms of accuracy and speed. The lightweight neural models used in the study are able to achieve state-of-the-art results in several emotion recognition and engagement detection tasks. The proposed framework can be integrated into existing e-learning tools for real-time assessment of students' emotions and comprehension, providing a valuable tool for teachers and educators. |
| **Country**: Rusia  **Year of Publication**:2022.  (Nikitin et al., 2022) | The aim of the research is the development of methods and algorithms for assessing the psycho-emotional state of learners and their involvement in the teaching process in online education, as well as evaluation of their impact on the efficiency of training. | More than 100 students of Russian State Agrarian University – Moscow Timiryazev Agricultural Academy and Financial University took part in the survey. The following characteristics were measured during the online teaching: involvement in the educational process, students’ psycho-emotional state. In order to build the training algorithms assessing students’ engagement in online learning, the modernised dataset DAiSEE was used; for assessing the psycho-emotional state – the dataset fer2013. Convolutional neural networks were used as model algorithms. The ROC curve and accuracy parameters were used as metrics for model training quality. The Farrar-Glauber test was used for the analysis of multicollinearity of parameters. To prove the efficiency of the developed methods, the statistical criteria – Fisher’s F-test and Student’s t-test – were used. | The developed models demonstrated an excellent quality of training. The accuracy of recognising student engagement in the learning process exceeded 90%, the accuracy of identifying the emotions in online learning was over 90%. The inculcation of the said algorithms in the online learning system showed due efficiency. It was proved that the control group (23 students) and the experimental group (25 students) differed significantly in statistical terms (temp = 2.53; p < 0.05). The learners’ involvement, their emotions and the actual knowledge of the subject area did not show any strong correlation with each other (FGemp=3.61 < FGcrit=7.81). |
| **Country**: India  **Year of Publication**: 2022  (Mehta et al., 2022) | This paper attempts to present a three-dimensional DenseNet self-attention neural network (DenseAttNet) used to identify and evaluate student participation in modern and traditional educational programs. | They used a database containing a sequence of videos of the students.  The proposed pipeline consists of multiple stages, including temporal down-sampling of video clip frames, face detection and alignment with Dlib, and prediction with the proposed neural network. The aligned face image is resized and concatenated to get 30×224×224×3-dimension image cube that is fed as input to the DenseAttNet model to predict affective states.  The methodological steps were as follows:   1. Pre-processing 2. DenseNet self-attention network (DenseAttNet) 3. Class-balanced (CB) los 4. Experimentation | With the Dataset for Affective States in E-Environments (DAiSEE), the proposed DenseAttNet model outperformed all other existing methods, achieving baseline accuracy of 63.59% for engagement classification and 54.27% for boredom classification, respectively.  Besides, DenseAttNet trained on all four multi-labels, namely boredom, engagement, confusion, and frustration has registered an accuracy of 81.17%, 94.85%, 90.96%, and 95.85%, respectively. In addition, we performed a regression experiment on DAiSEE and obtained the lowest Mean Square Error (MSE) value of 0.0347.  Finally, the proposed approach achieves a competitive MSE of 0.0877 when validated on the Emotion Recognition in the Wild Engagement Prediction (EmotiW-EP) dataset.  In future work, we will explore more efficient deep learning algorithms and advanced loss functions that can account for data imbalances and multi-class multi-label emotion categorization in DAiSEE. |
| **Country**: India.  **Year of Publication**: 2022.  (Jagadeesh & Baranidharan, 2022) | To determine which facial physical behaviours are associated with emotional states and then to determine how these emotional states are related to student understanding. | The sample or participants in this study are online learners whose facial expressions were captured in real-time videos. The dataset used for the study was gathered from real-time online learning videos, and several frames were accessed from each video. The benchmark data used for the experiment was collected from a website and consisted of 48 videos, each with 1000 frames. The total number of video frames used for experimentation was 100, and each video consisted of 50 frames. The frames from the acquired videos were used as input for the suggested model. Therefore, the participants in this study were online learners whose facial expressions were captured in real-time videos. | The study conducted an analysis of distinct heuristic-based algorithms for online learning FER models, evaluating metrics such as Accuracy, Sensitivity, Specificity, Precision, FPR, FNR, NPV, FDR, F1 Score, and MCC.  The performance of the online learning FER model was assessed using benchmark datasets and various deep structure learning methods. Metrics such as Accuracy, Sensitivity, Specificity, Precision, FPR, FNR, NPV, FDR, F1 Score, and MCC were considered in the analysis.  The outcomes of the GUI implementation for the suggested online learning FER model were presented, showcasing the user interface design and functionality.  The study demonstrated the effectiveness of the novel deep learning model in facial expression recognition of online learners from real-time videos, providing insights into the potential applications of emotion detection technologies in virtual learning environments. |
| **Country**: Thailand.  **Year of Publication**: 2022.  (Wetcho & Na-Songkhla, 2022) | The study aims to explore the ownership and usage of technological devices among pre-service teachers, investigate the signals and elements used for emotion recognition, and analyze the potential of mobile and wearable devices in supporting emotion regulation and social sharing of emotion in educational settings. | The study conducted on pre-service teachers using mobile and wearable devices for emotion recognition and social sharing of emotion to support emotion regulation in mCSCL environments employed a quantitative approach. The participants, pre-service teachers from three universities in Thailand, were selected through convenience sampling. Data was collected via an online survey, and a final sample of 183 participants was included in the study. The survey included questions related to emotion recognition tools, emotion regulation, social sharing of emotion, and the use of mCSCL applications. Various validated scales and questionnaires were adapted to assess these constructs, including náli on emotion recognition, emotion regulation, and social sharing of emotion.  The study also Involved regression análisis to examine the predictive relationships between emotion recognition, social sharing of emotion, and emotion regulation. The research methodology aimed to investigate the role of technology in supporting emotional aspects of learning and collaboration among pre-service teachers. | Overall, the study provided insights into the use of technology for emotion-related activities among pre-service teachers and emphasized the importance of emotional skills in educational settings.  Most pre-service teachers owned smartphones, followed by PCs/laptops, with a notable increase in the use of Bluetooth earphones. Wearable devices were less commonly owned, with smartwatches being the most prevalent among participants. Participants predominantly relied on physical signals such as expressions, voice/speech, and gesture/posture for recognizing emotions in themselves and others.  Participants predominantly relied on physical signals such as expressions, voice/speech, and gesture/posture for recognizing emotions in themselves and others.  Pre-service teachers engaged in activities like listening, speaking, and face-to-face communication to adjust their emotions. Networking, cooperation, and collaboration were identified as important factors in emotion regulation.  The study highlighted the significance of emotion recognition, social sharing of emotion, and emotion regulation in mCSCL environments, emphasizing their role in supporting socio-affective factors in teacher education development.  The research identified the need for further exploration of emotion interaction with cognitive perspectives, cultural considerations, and scientific validation in emotion detection technologies. |
| **Country**: Ecuador  **Year of Publication**: 2023  (Villegas-Ch et al., 2023). | This work applies techniques for the recognition and processing of facial gestures and the classification of emotions focused on learning. | The data capture is carried out in two stages, in which the first 20 students participate, of which 12 are men and 8 women, with an age range of 18 to 20 years. In the second stage, 18 students participate, of which 10 are men and 8 women, who are in the same age range.  **Total**: 38 students  For the development of the method, three fundamental bases are considered, image databases, affective computing, and emotion recognition systems with artificial intelligence. These bases guarantee the functioning of the identification of the emotions of the students, through the gestures that their faces generate in a didactic environment. | This system helps the tutor in a modality of face-toface education and allows him to evaluate emotional aspects and not only cognitive ones.  The gesture recognition system for the identification of emotions allows the teacher to obtain an additional variable, with which it is possible to improve the teaching method. According to the results obtained, it has been identified that students generate a variety of gestures during the teaching process, and these are generally linked to a certain emotion. By identifying the emotions of the student during a teaching process, the teacher provides feedback on her method and can make decisions that allow him to improve the teaching environment.  In the training of the neural network, a validation accuracy percentage of over 70% has been achieved, this percentage is adequate considering the results found in similar works that have used other platforms.  Among the positive characteristics of the developed model, it can be established that generating the training with the students’ images, allows for improved recognition of gestures.  In the validation of results, it is recommended to make a comparison of the emotion recognition system with a greater number of sessions. In addition, it is important to include a greater variety of gender in the group, as well as to include in the study people who are in a higher age range than the one included in the study.  AI algorithms can have a hard time understanding the context in which an emotion occurs. Without context, algorithms can misinterpret emotions and provide incorrect results. Often people experience multiple emotions simultaneously, which can make it challenging to detect accurate emotions. AI algorithms may also have difficulty detecting mixed emotions and may provide inaccurate or conflicting results. Furthermore, emotions and their ways of expression can vary between cultures and geographic regions. Therefore, AI algorithms designed to detect emotion in college students may not be accurate across cultures. |
| **Country**: Egypt.  **Year of Publication**: 2023.  (Aly, & Fathi, 2023). | To present an online educational platform that utilizes facial expression recognition technology to monitor students' progress within the classroom. The system captures images of students, processes the facial data, and assesses students' learning statuses using expression recognition techniques. | Improving the network structure of expression recognition by integrating the Convolutional Block Attention Mechanism (CBAM) and refining residual modules.  Developing an architecture for an online learning status monitoring system that incorporates the enhanced expression recognition methodology and CBAM attention mechanism.  Assessing online learning status by monitoring students' emotional states, evaluating classroom dynamics, and providing relevant cues to aid educators in overseeing students' progress during online learning.  The study focuses on enhancing the precision of expression recognition, refining teaching strategies, and assessing students' engagement within the online classroom setting. | Improved Facial Expression Recognition Accuracy: Utilization of ResNet-50 for effective feature extraction led to enhanced facial expression recognition accuracy.  Adjustment of the residual down-sampling module improved the correlation among input features, reducing the loss of feature information  Real-Time Performance Evaluation: The proposed model exhibited reduced time complexity, achieving frames per second (fps) of 32 on GPU, 14 on CPU, and 19 on the Jetson Nano device.  Comparative analysis on the CK+ dataset showed competitive accuracy and computational efficiency, with a test accuracy of 94.58% and a run-time of 61 seconds  Comparative Analysis Against State-of-the-Art Techniques: Experimental results demonstrated that the refined ResNet model surpassed alternative methods in enhancing expression classification accuracy, particularly within the RAF-DB dataset. The proposed model achieved a balance between accuracy and run-time, making it suitable for real-world applications on edge devices.  The study's results highlight the effectiveness of the enhanced deep learning model in improving facial expression recognition accuracy and real-time performance, showcasing its potential for practical deployment in online learning environments. |
| **Country**: India.  **Year of Publication**: 2023.  (Lokare & Jadhav, 2024) | This study presents an AI-based learning prediction model that effectively predicts learning styles based on input attributes such as attention, meditation, cognitive workload, facial expressions, and emotional state. | This research proposes a prediction model based on attention, meditation, cognitive workload, facial expression., and emotional state for the four learning styles: visual, aural, read-write, and kinesthetic. By analyzing these five factors, the model can predict which learning style would be most effective for a particular student.  The study involved 52 students who participated in EEG data collection while performing four equally difficult tasks. These tasks were chosen to cater to different learning styles. | An AI-based learning prediction model developed using input attributes such as attention, meditation, cognitive workload, facial expressions, and emotional state effectively predicts learning styles.  There are several advantages to using a learning styles prediction model, such as personalizing the learning experience for students, helping students understand their learning styles, and enabling institutions to tailor their teaching methods and resources. Overall, the model provides valuable insights into how individuals learn, leading to more effective and personalized learning experiences for students. AI-based Learning Style Prediction Model represents a significant step toward realizing personalized and effective learning. By harnessing the power of artificial intelligence, it empowers educators to tailor educational experiences to learners’ _individual needs and preferences, ultimately fostering a more engaging and successful learning journey for all. |
| **Country**: India.  **Year of Publication**: 2023.  (Begum, Neelima & Valan 2023). | To develop an emotion recognition system specifically tailored for E-learning environments based on facial expressions. The researchers aim to address the challenge of detecting emotions like Confusion, Boredom, Concentration, and Self-Confidence in learners engaged in online education. | Research study that presents a new method for emotion recognition in E-learning environments based on facial expressions. The study involves proposing a novel approach, conducting experiments, and analyzing the results to evaluate the performance of the proposed system. The focus is on developing a system that can accurately classify emotions specific to E-learning environments, such as Confusion, Boredom, Concentration, and Self-Confidence, with a high level of accuracy.  **Input Image**: Face images capturing various facial gestures of different individuals expressing emotions like Confused, Boredom, Concentration, and Self-Confidence are used as input to the model.  **Pre-processing**: The input image is resized to a standard size of 200 x 300 using bilinear interpolation. A Gaussian low pass filter is applied to the resized image to enhance image quality and remove noise.  **Feature Extraction**: Multiple portions of the face, including eyes, eyebrows, eyelids, forehead, mouth, and chin, are considered for feature extraction using local binary patterns (LBP).  **Classification**: A fuzzy neural network is employed for classifying the extracted features into emotion categories like Confused, Boredom, Concentration, and Self-Confidence. | The proposed method utilizes the Viola–Jones algorithm for face detection, local binary patterns (LBP) for feature extraction, and a fuzzy neural network for classification. The system considers multiple portions of the face to classify emotions like Confusion, Boredom, Concentration, and Self-Confidence.  After training the dataset with different epochs (10, 30, 50, 100) and varying numbers of bins (4 bins, 8 bins, 16 bins), an optimized result was obtained with 50 epochs and 8 bins (32 bin size). This configuration achieved a prediction accuracy of approximately 91.25%.  The proposed system outperformed existing methods by accurately recognizing and classifying emotions specific to E-learning environments. The high prediction accuracy of 91.25% demonstrates the effectiveness of the system in detecting emotions like Confusion, Boredom, Concentration, and Self-Confidence from facial expressions.  The study showcases the potential of the developed emotion recognition system to enhance the quality of teaching and learning in E-learning environments by accurately interpreting learner emotions. |
| **Country**: India  **Year of Publication**: 2023  (Yugal et al., 2023) | In this research SVM, MCC, NLP and CNN machine learning algorithms are applied to detect students’ feelings and emotions to track the feedback via IoT enabled system | To achieve this goal with IoT-enabled objects, an academic can create a more personalized and effective learning environment for students, trainees and interns. A novel framework emulated with IoT and machine learning techniques is designed and implemented to analyze student performance. The model receives feedback from 1000 students using IoT devices and analyzes missing information in learning systems, that missing information lacked effective learning. This emulated framework analyzes the performance of the model. A novel and innovative early warning system is also created to send the warning via WhatsApp or email to multiple users at once, upon reaching certain targets such as file size limits, etc. | The development of an affective model for emotion detection and sentiment analysis in learner feedback brings numerous benefits to enhancing educational experiences. By accurately detecting and analyzing emotions and sentiments expressed by learners, the affective model provides valuable insights to educators and instructional designers. These insights enable them to personalize instruction, address learner challenges, and create a supportive and engaging learning environment. One significant benefit is the ability to tailor teaching strategies to meet the specific needs of learners. By understanding learners’ emotional states, educators can adapt their instruction to provide appropriate support, motivation, and challenges. This personalized approach helps improve learner engagement, motivation, and overall performance. This research enables educators to identify areas of learner satisfaction or dissatisfaction with learning materials and to analyze sentiments expressed in feedback to help identify strengths and weaknesses in instructional design, allowing for targeted improvements. By addressing areas of dissatisfaction, educators can enhance the quality of learning materials and create a positive learning experience for learners.  In recent years, most of the research exhibits in the field of Education 4.0 Training Systems (ETS) and Industry 4.0 Training Systems (ITS) that has the ability to learn the behavior of the learners, interns, or trainees. Understanding the feelings and emotions of learners toward learning is essential for creating a practical and exciting learning experience. Patience-emotions detection and sentiments analysis have emerged as an integral part of the understanding of the behaviors of learners, thus there is a need to expand the overall educational or training process in academics and industries. |
| **Country**: Iran  **Year of Publication**: 2023  (Moghadam et al., 2023) | In this paper, an AI-based decision framework is proposed and implemented for e-learning systems that identify suitable micro-brake activities based on the learner’s emotional state through an evolutionary genetic algorithm to change learner’s mood and increase learning performance. | The participant of this study were forty teenage students (ranging from 10 to 15 years old learners, average = 12.3) enrolled in a general English course. These students came from 2 classes of 20 that were randomly selected from a pool of 17 classes studying at the language centre and they were randomly assigned to control and experimental groups. Each class had a mix of male and female students (see Table 1 for more detail). These participants were recruited based on the number of classes available to the researchers. Care was taken that the sample size does not negatively impact the reliability or validity of the results (more on this later in the section). The students of the experimental group (Group A) were taught using our proposed framework and they had access to break activities, while in the control group (Group B); the students were still taught using the same framework but they did not have access to break activities. These break activities were suggested to students by the system, based on their preferences and self-reported emotional state. The process of how the system selected activities will be explained in the next section.  The period of intervention was one semester and the learners’ performance was tested in terms of their ability in the learning of English before and after the intervention. The pre and post-test consisted of 40 multiple-choice questions, and 10 fill in the blank questions and were all of equal weight. An independent-samples t-test was deemed suitable for the purpose of this study and to compare the pre and post-test exam marks, for the control and experimental group. | The results of this study demonstrated the importance of learners’ emotions in their learning performance and proved the effectiveness of our proposed framework and the success of the recommended micro-break activities chosen based on learners’ emotions and preferences. The findings of this study have important practical implications in designing adaptive e-learning systems and learning management systems such as Moodle. They also contribute to theoretical implications in the field of AI and learner emotions by suggesting a novel approach to identifying, categorizing, and offering a learning path that can cater to the needs of individual learners. |
| **Country**: Japan.  **Year of Publication**: 2023.  (Lasri, Riadsolh & Elbelkacemi, 2023). | To investigate the use of deep learning techniques, specifically deep convolutional neural networks (DCNN), for recognizing facial emotions of deaf and hard-of-hearing students. | The methodology employed in the study involves utilizing a deep convolutional neural network (DCNN) model, specifically the VGG-16 model, for facial emotion recognition of deaf and hard-of-hearing students. Transfer learning techniques are applied to fine-tune the pre-trained VGG-16 model on two facial image datasets: the Japanese female facial expression (JAFFE) dataset and the Karolinska directed emotional faces (KDEF) dataset. The performance of the proposed model is then evaluated and compared to seven different pre-trained DCNN models, including VGG-19, Inception v3, DenseNet-121, DenseNet-161, MobileNet, ResNet-50, and Xception, on the JAFFE and KDEF datasets. The study aims to detect the engagement levels of deaf and hard-of-hearing students based on their facial expressions in real-time.  The sample in this study consists of facial images of deaf and hard-of-hearing students captured using a camera in a classroom setting. These images are used to train and test the deep learning model for facial emotion recognition and engagement detection. The JAFFE and KDEF datasets are specifically chosen for training and fine-tuning the VGG-16 model, with comparisons made against other pre-trained DCNN models to evaluate the performance of the proposed approach. | The VGG-16 model achieved the best classification accuracy of 97.7% and 86.33% for the JAFFE and KDEF datasets, respectively, when using na 80-20% split for validation. In the 10-fold cross-validation case, the VGG-16 model achieved even higher classification accuracies of 98% and 99% for the JAFFE and KDEF datasets, respectively.  The proposed approach with the VGG-16 model outperformed other state-of-the-art methods for facial emotion recognition and engagement detection of deaf and hard-of-hearing students. The system successfully identified different engagement levels ("highly engaged," "nominally engaged," and "not engaged") based on facial expressions captured in a classroom environment.  The study demonstrated the potential of using deep convolutional neural networks and transfer learning techniques to accurately detect engagement levels of deaf and hard-of-hearing students from their facial emotions. The proposed system could help teachers adapt teaching material based on individual student engagement levels, ultimately improving classroom management and educational outcomes for these students.  The results suggest that the developed system shows promise in effectively recognizing and categorizing engagement levels of deaf and hard-of-hearing students based on their facial expressions, paving the way for enhanced educational experiences in classroom settings. |
| **Country**: Jordan  **Year of Publication**: 2023  (Qaqish et al., 2023) | This study investigated the Jordanian community’s perspectives and feelings on the transition from pure face-to-face education to blended education by examining related tweets in the post-COVID era. Specifically, using NLP Emotion detection and Sentiment Analysis approaches, as well as deep learning models. | The dataset consisted of 4000 tweets in Arabic Language; each tweet was made up of Arabic words, English words, Emojis, Special characters, and Punctuation marks. Where the Tweets were divided into three categories based on people’s opinions about the blended education: 800 with the blended education, 1300 against blended education, and the remaining 1900 with both categories’ hashtags representing the neutral group | As a result of analyzing the collected tweets, 18.75% of studied Jordanian’s community sample are dissatisfied (Anger and Hate), 21.25% are negative (Sad), 13% are Happy, and 24.50 percent are Neutral about it  Finally, in addition to the five emotions explored in this study, we will concentrate on recognizing the commonly unnoticed emotions such as frustration and impatience. |
| **Country**: Mexico  **Year of Publication**: 2023  (Cárdenas-López et al., 2023) | This paper presents three different fusion methods applied to three image- and text-based datasets for learning-centered emotion recognition. | Using some conventional deep learning architectures, the three new multimodal datasets showed promising results compared to similar architectures trained on unimodal information. | The improvement of one of the methods (embedding-based representation) was 4% compared to single modality hyperparameter optimization. The main objective of this study is to compare the feasibility of semantic fusion of learning-centric multimodal emotional data from different datasets for intelligent tutoring system applications. |
| **Country**: Spain  **Year of Publication**: 2023  (Fernández Herrero et al., 2023) | To test the suitability of an automatic emotional management system in the classroom following the framework of the control-value theory of achievement emotions (CVT), we evaluate the performance of an emotional expression recognition software of our creation in a synchronous online context. | 60 students from the Faculty of Education at the University of Alicante participated in 16 educational activities by recording close-ups of their faces and completing the AEQ emotional self-report, as well as detailed reports of the subsequent review of their videos. In addition, they completed the VCQ-36 test to measure their volitional competencies and relate their influence on their emotional response. | The results indicate high consistency between the emotional expressions detected by the automatic system and the detailed emotional self-reports, but insufficient accuracy to meet CVT requirements. On the other hand, both the results of the AEQ test and the emotion expression recognition software suggest students' preference for participatory versus passive activities. Meanwhile, the results of the statistical analysis indicate that volitional competencies seem to influence students' emotional response in the educational context, although the AI system does not show sufficient sensitivity in this domain.  Deep learning technology based on convolutional neural networks powers automatic systems focused on facial expression recognition from image analysis. What this article adds There is high consistency between the emotional expressions detected by the AI system and students' emotional self-reports, but the AI system provides only emotional valences, insufficient to meet the control-value theory of achievement emotions (CVT) framework.  Only if sensitivity were improved could a real-time, easy-to-interpret emotional expression recognition software interface be implemented to assist teachers with emotional management of their classes within the CVT framework, maximizing their motivation and engagement. |
| **Country**: United Arab Emirates  **Year of Publication**: 2023  (Trabelsi et al., 2023) | This study harnesses modern technology to introduce an intelligent real-time vision-based classroom to monitor students’ emotions, attendance, and attention levels even when they have face masks on. | They used a machine learning approach to train students’ behavior recognition models, including identifying facial expressions, to identify students’ attention/non-attention in a classroom. The attention/no-attention dataset is collected based on nine categories. The dataset is given the YOLOv5 pre-trained weights for training. For validation, the performance of various versions of the YOLOv5 model (v5m, v5n, v5l, v5s, and v5x) are compared based on different evaluation measures (precision, recall, mAP, and F1 score). | The results show that all models show promising performance with 76% average accuracy. Applying the developed model can enable instructors to visualize students’ behavior and emotional states at different levels, allowing them to appropriately manage teaching sessions by considering student-centered learning scenarios. Overall, the proposed model will enhance instructors’ performance and students at an academic level. Associating students’ emotions with their actions can enhance the accuracy of attention recognition.  The proposed system, which utilizes deep learning algorithms, has been successfully tested on a small group of seven students, and the results have been promising. The system can automatically monitor students’ behavioral and emotional patterns, which, in turn, assists educators in assessing students’ attention levels. It also serves as a decision-making assistant, providing strategic information to educators in real-time and offline, by detecting student behavior, emotions, attendance, and progress statistics. We have implemented the deep learning algorithm YOLOv5 [45,54], known for its efficient accuracy and faster detection speed compared to other variants such as YOLOv7 and YOLOv8.  However, we acknowledge that there are challenges to overcome, such as the need for a larger dataset, which is essential for deep learning neural networks to work effectively. The system will continue learning and improving using AI and machine learning [55]. As part of our future research, we plan to integrate the action/behavior and emotion models into social robots to act as teaching assistants, which can reach out to less attentive students and make them more interactive. |
| **Country**: United States.  **Year of Publication**: 2023.  (Dehbozorgi & Kunuku, 2023) | To analyze the correlation between students’ emotional states, the topics they discuss in teams, and their academic performance. | The researchers conducted a study where they recorded the speeches of 38 students during five class sessions, resulting in a total of 95 discussion records. The students were assessed through tests, assignments, and class activities, with their final grades used as the performance metric. The recorded speech files were processed through a developed pipeline to extract emotions and topics discussed. The model identified 40 different emotion classes and categorized topics as course-related or non-related for analysis. The relationship between expressed emotions, discussed topics, and students’ performance was analyzed using the Pearson correlation. | The study found a significant positive correlation between students' positive emotions, such as "relief" and "satisfaction," and their grades, indicating a direct link between emotional well-being and academic performance. Students who dedicated their teamwork time to discussing course-related topics performed better in completing class activities on time and overall academic performance. Additionally, there was a strong positive correlation between course-related topics discussed in teams and grades, while a negative correlation was observed between non-course-related topics and grades. These findings suggest that focusing on course-related discussions and fostering positive emotions among team members can lead to improved academic success. |
